# Microphysiological system for studying contractile differences in young, active, and old, sedentary adult derived skeletal muscle cells

**DOI:** 10.1111/acel.13650

**Published:** 2022-06-02

**Authors:** Shelby Giza, Jorge A. Mojica‐Santiago, Maddalena Parafati, Legrand K. Malany, Don Platt, Christine E. Schmidt, Paul M. Coen, Siobhan Malany

**Affiliations:** ^1^ Department of Pharmacodynamics, College of Pharmacy University of Florida Gainesville Florida USA; ^2^ J. Crayton Pruitt Family Department of Biomedical Engineering, Herbert Wertheim College of Engineering University of Florida Gainesville Florida USA; ^3^ Micro‐gRx, INC Orlando Florida USA; ^4^ Micro Aerospace Solutions Melbourne Florida USA; ^5^ Translational Research Institute AdventHealth Orlando Florida USA

**Keywords:** bioengineered skeletal muscle, cellular electrical stimulation, human CD56^+^ primary cells, muscle myogenesis gene expression, sarcomere immunofluorescence

## Abstract

Microphysiological systems (MPS), also referred to as tissue chips, incorporating 3D skeletal myobundles are a novel approach for physiological and pharmacological studies to uncover new medical treatments for sarcopenia. We characterize a MPS in which engineered skeletal muscle myobundles derived from donor‐specific satellite cells that model aged phenotypes are encapsulated in a perfused tissue chip platform containing platinum electrodes. Our myobundles were derived from CD56^+^ myogenic cells obtained via percutaneous biopsy of the vastus lateralis from adults phenotyped by age and physical activity. Following 17 days differentiation including 5 days of a 3 V, 2 Hz electrical stimulation regime, the myobundles exhibited fused myotube alignment and upregulation of myogenic, myofiber assembly, signaling and contractile genes as demonstrated by gene array profiling and localization of key components of the sarcomere. Our results demonstrate that myobundles derived from the young, active (YA) group showed high intensity immunofluorescent staining of α‐actinin proteins and responded to electrical stimuli with a ~1 μm displacement magnitude compared with non‐stimulated myobundles. Myobundles derived from older sedentary group (OS) did not display a synchronous contraction response. Hypertrophic potential is increased in YA‐derived myobundles in response to stimulation as shown by upregulation of insulin growth factor (IGF‐1), α‐actinin (ACTN3, ACTA1) and fast twitch troponin protein (TNNI2) compared with OS‐derived myobundles. Our MPS mimics disease states of muscle decline and thus provides an aged system and experimental platform to investigate electrical stimulation mimicking exercise regimes and may be adapted to long duration studies of compound efficacy and toxicity for therapeutic evaluation against sarcopenia.

## Graphical abstract

Novel platform to study age‐related differences in skeletal muscle response to electrical stimulation using a 3 dimensional microphysiological system D MPS that incorporates cells derived from well phenotyped adults characterized by age and physical activity.

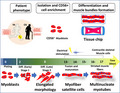

**Abbreviations**
CT: cycle threshold
DAC: digital to analog converter
DC: direct current
DEG: differentially expressed genes
DMSO: dimethyl sulfoxide
DIC: digital image correlation
E‐stim: electrical stimulation
FACS: fluorescence activated cell sorting
FC: fold change
GUI: graphic user interface
GO: gene ontology
HZ: Hertz
MACS: magnetic‐activated cell sorting
MEMS: microelectromechanical systems
MPS: microphysiological Systems
non‐stim: no electrical stimulation
OS: old sedentary
PDMS: polydimethylsiloxane
RNA: ribonucleic acid
SKM: skeletal muscle
YA: young active

## 1 INTRODUCTION

The segment of the population aged 65 and older is rapidly expanding and (United Nations, Department of Economic and Social Affairs, Population Division, 2019) represents an enormous healthcare challenge as old age is a risk factor for many chronic diseases (Atella et al., 2019). Sarcopenia is a progressive pathology characterized by the loss of muscle mass and strength, and is prevalent in those over 60 years old (Papadopoulou, 2020). Sarcopenia eventually leads to loss of independence and elevated risk of morbidity and mortality and is a massive economic burden with direct annual healthcare costs estimated to be over $19 billion (Goates et al., 2019). Despite these medical and socioeconomic costs, there is a lack of effective therapeutic options available in part because the mechanisms underlying sarcopenia are challenging to study over many years in the same patient.

Satellite cells are the predominant stem cell population in adult skeletal muscle and are thought to play a role in the progressive pathology of sarcopenia (Alway, Myers, & Mohamed, 2014). Satellite cell content is decreased in muscles of older people humans (Verdijk et al., 2014), and there is evidence of functional impairment due in part to altered systemic factors that impact satellite cell activity and differentiation (Conboy & Rando, 2005). Cell‐autonomous alterations also contribute to the functional deficit of old satellite cells (Brack & Muñoz‐Cánoves, 2016). In addition, age and elevated levels of physical activity have been shown to reprogram muscle satellite cells such that they retain key aspects of the donor's phenotype once in culture. For example, muscle cells from physically active or trained donors retain high oxidative capacity (Bourlier et al., 2013; Lund, Helle, et al., 2018a; Lund, S Tangen, et al., 2018b; Pino et al., 2019) and for older adults, muscle cells have lower oxidative capacity and fewer cells with lower capacity for proliferation and differentiation (Aas, Thoresen, Rustan, & Lund, 2020; Chen, Datzkiw, & Rudnicki, 2020; Sousa‐Victor et al., 2014). As such, primary human muscle satellite cells represent a valuable in vitro model to study aging and the impact of exercise.

Human skeletal muscle 3D microphysiological systems (MPS), also referred to as tissue chips, that mimic muscle morphology and function on a tissue level, hold promise as therapeutic testing platforms. There have been significant advancements in development of MPS devices incorporating myogenic cells reviewed in the literature (Madden, Juhas, Kraus, Truskey, & Bursac, 2015). Muscle myobundles have likewise been generated to polymerize around deformable micro posts (Agrawal, Aung, & Varghese, 2017; Mills et al., 2019; Vandenburgh et al., 2008). In these systems, skeletal muscle cells have been embedded in various naturally derived hydrogels to mimic extracellular matrix (ECM) to enhance maintenance and repair of skeletal muscle and aid in myobundle force transmission (Csapo, Gumpenberger, & Wessner, 2020; Hinds, Bian, Dennis, & Bursac, 2011). Introduction of electrode wires and microelectrode or optical sensors allows for electrical stimulation of the muscle cells and recording of the contractile behavior via time‐lapsed microscopy or force calculations (Truskey, 2018). Electrochemical stimulation coupled with mechanical contraction of skeletal muscle bundles, aimed at mimicking the action of the motor neurons, has a pivotal role in inducing changes in cell morphology, hypertrophy, and maturation (Guo, Cheung, Yeung, Zhang, & Yeung, 2012; Jaatinen et al., 2016; Langelaan et al., 2011).

These 3D MPS have the advantage to be more physiologically relevant in studying biomechanics than 2D muscle cultures and hold the promise to understand disease mechanisms and tissue remodeling under controlled conditions. However, few studies till date have been published that use human primary cells in a microfluidic environment or use donor‐specific cells, to model a disease state, such as from older, sedentary adults who are at higher risk for developing age‐related muscle diseases. Moreover, contractile characteristics of muscle cells from populations of old and young donors have also not been extensively studied. Physiology and pharmacological studies in MPS using donor‐specific cells may result in a greater likelihood of successful clinical trials of drug candidates. In this respect, the development of tissue chips incorporating satellite cells from younger and older adult donors capable of accurately modeling both healthy and atrophied states would accelerate the pace of pharmacological studies and contribute to the advancement of tissue engineering.

Herein, we report on validation studies of a microfluidic MPS that demonstrates contractile bioengineered myobundles derived from muscle biopsies from young, athletic (YA) and older, sedentary (OS) adults. The goal of our study is to develop a model of muscle aging to investigate the cellular mechanisms underlying sarcopenia, to use for pre‐clinical drug evaluation and to inform our studies being conducted on the International Space Station to study effects of microgravity‐induced muscle atrophy that may mimic the physiological effects of aging on a faster timescale than on Earth (Sharma et al., 2022). We reasoned that isolating muscle cells from these two distinct human populations maximized our chance of working with phenotypically distinct muscle cells. Thus, incorporating muscle precursor cells isolated from adults phenotyped based on age and physical activity as an autonomous cell model to measure intrinsic effects of cellular stress offers the possibility of studying innate characteristics of the donor and more accurately reflects human physiology disease states. In this respect, our MPS seeks to be a more effective bridge between discovery and clinical research.

## 2 RESULTS

### 2.1 Human skeletal muscle cell young active (YA) and old sedentary (OS) phenotypes

Ten male participants were screened and enrolled to the following groups: young active (YA; 21–40 years, *n* = 5) and older sedentary (OS, 65–90 years, *n* = 5). Participants were considered active if they engaged in endurance exercise (running, cycling, or swimming) at least 3 days a week without extensive lay off over the previous 6 months. Participants were considered sedentary if they completed one or fewer structured exercise sessions a week. The characteristics of the study groups are shown in Table 1. As per the study design, the YA group participants were younger, leaner, had a greater cardiorespiratory fitness, and lower BMI compared with the OS group indicating higher fitness and lower adiposity typically associated with an endurance‐trained physically active lifestyle. Muscle progenitor cells were isolated from a 50–100 mg biopsy specimen from these volunteers using a pre‐plate technique previously described (Sparks et al., 2011). The cells from each donor were expanded for two passages, counted, and pooled in equal ratios to provide mean YA and mean OS myoblast stocks referred to as YA cohort and OS cohort. The pooled myoblasts were grown to confluence, aliquoted, and frozen. CD56 is an important cell surface marker known to be expressed by satellite cells and other pro‐myogenic cells within skeletal muscle (Vauchez et al., 2009). Prior to cell seeding into tissue chips, thawed aliquots were purified to enrich for CD56^+^ myogenic cells. The percent recovery of CD56^+^ cells compared with the total cell count prior to purification was calculated as 66 ± 15% for YA (*n* = 5 aliquots) and 50 ± 10% for OS (*n* = 5 aliquots) cohorts. Enrichment of the CD56^+^ myogenic cell pools was confirmed by flow cytometry and determined to be >99% CD56^+^ (Figure S1).

**TABLE 1:** Human subject characteristics

	Young, active (male, *n* = 5)	Old, sedentary (male, *n* = 5)	*p* Value*
Age, years	35 ± 4	70 ± 4	<0.001
Weight, Kg	70 ± 4	87 ± 14	0.01
BMI, Kg/m^2^	22 ± 2	28 ± 4	0.001
Total fat mass, Kg	10.8 ± 3.9	30.2 ± 8.8	0.01
Total lean mass, Kg	60.4 ± 3.3	57.1 ± 5.9	0.01
VO peak, L/min	4.1 ± 0.4	1.8 ± 0.2	<0.001
VO_2_ peak, L/kg/min	58.6 ± 5	21.4 ± 2.7	<0.001

*Note:* Values are presented as mean ± SD.

Abbreviations: BMI, body mass index; VO_2_ peak, maximum rate of oxygen consumption.

* Group differences were determined using a non‐paired students *t*‐test.

### 2.2 YA‐ and OS‐derived myobundles display varying multinucleation

**FIGURE 1:**
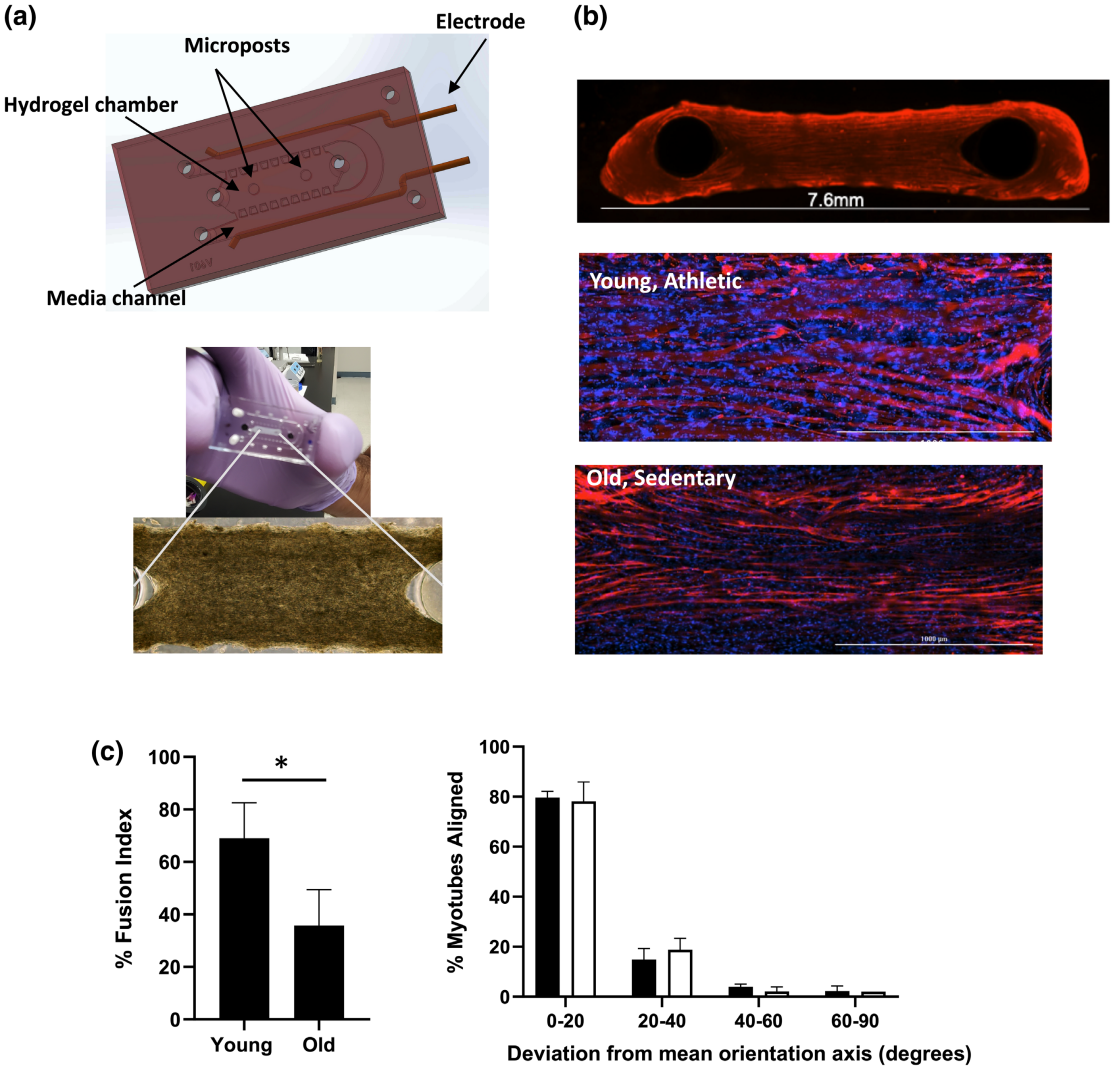
Characterization of YA‐ and OS‐derived muscle myobundles in microfluidic chip. a upper) Computer‐assisted design rendering of microfluidic PDMS chip indicating where the myobundles are formed around the two posts and a lower) a representative phase contract image of myobundle formation after 2 days in culture. b top) Representative (4×) image of OS‐derived myobundle after differentiation phase and immunofluorescence stained with MF‐20 (red) and b middle) Immunostaining with MF‐20 (red) and Dapi (blue) of YA‐derived myobundle and b bottom) OS‐derived myobundles (20×, Scale bar 1000 μm). c left) Percent fusion index determined by immunostaining shown in b and spot counting algorithm. Data are average ± standard deviation of seven chips per pooled cell cohort (*p < 0.05 analyzed by two‐tailed paired *t*‐test). c right) Mean alignment score showing the deviation of the longitudinal myotube axis from the mean orientation axis (line between posts) for YA‐derived myobundles (black bars) and OS‐derived myobundles (white bars). Values are calculated as a percent of total myotubes per sample. Data average of three individual chips per cohort

The CD56^+^ enriched cells were encapsulated into a collagen‐Matrigel 3D scaffold to provide a suitable tissue‐mimicking microenvironment for the bioengineered skeletal muscle myobundles. The cell‐laden hydrogel was seeded into an enclosed microfluidic chip fabricated from PDMS and bonded to glass. Platinum electrodes were embedded along the media channel and extended 3.2 mm outside the PDMS for attachment to connectors and a pulse generator during electrical stimulation (Figure 1a, upper). The PDMS chip featured an inner channel 3.2 mm wide aligned with tapered surface‐tension pins set 0.3 mm apart and two PDMS posts of 1 mm diameter spaced 5 mm apart (center to center) to allow cells to condense around the posts and form a single myobundle per chip. The outer media channel allowed perfusion of the condensed myobundle. This 1:5 diameter to distance ratio described previously (Agrawal et al., 2017) proved to be optimal to form approximately 0.8–1 mm thick, stretched myobundles after 48 h when YA‐derived cells were seeded at 15 × 10^6^/mL and OS‐derived cells were seeded at 20 × 10^6^/mL as visualized by phase contrast microscopy (Figure 1a, lower). A higher density of YA cells caused too much strain and resulted in myobundles falling off the posts, whereas the OS cells at the lower density were too thin and did not condense well.

Myobundles were differentiated over an additional 12 days (Day 14) by a two‐step differentiation protocol and an intermittent flow rate to result in 3D myobundles 7.6 mm in length (Figure 1b, top). Myotube fusion index was determined by fluorescent immunostaining of myosin heavy chain (MF‐20) antibody, to identify differentiated from undifferentiated myotubes, and DAPI nuclear stain using a custom spot counting algorithm as described in Materials and Methods (Figure 1b, middle and bottom). The YA‐derived myobundles exhibited significantly higher multinucleated myotubes with a fusion index of 69 ± 14% compared with the OS‐derived myobundles with a fusion index of 36 ± 14% (Figure 1c left). Myotubes were also assigned an alignment score based on the angle of deviation from the mean horizontal line connecting the PDMS posts. Alignment scores were similar between the two cohorts at approximately 80% myotubes deviating <20 degrees from the mean horizontal axis (Figure 1c, right).

### 2.3 Direct current simulations confirm uniform electric field applied to myobundles

To assist in selecting an optimal range of applied voltage, based on electrodes located along the media channels parallel to the myobundle, finite element analyses of electric currents within the chip were conducted in COMSOL Multiphysics®. Electrochemical impedance spectroscopy (EIS) assessments of the tissue chips immersed with differentiation media were used to obtain the conductivity of the fluid and engineered tissue present between the electrodes. This parameter was used in the simulation tests of direct current (DC) electricity traveling through an electrolytic fluid (culture medium) alone or through the fluid and a porous hydrogel with a voltage range of 10 mV to 10 V. The resultant model, shown in Figure S2, predicted that a homogeneous distribution of electric field intensity of 9.0 ± 0.9 V/cm occurred at the horizontal centerline of the gel chamber between the tissue posts when a 2 V potential is applied (Figure S2a). When a 3 V potential is applied, a moderate increase in electric field strength of 13.3 ± 0.1 V/cm was predicted with a similar distribution of currents as 2 V (Figure S2b). Simulating at a higher voltage of 10 V resulted in a 41.3–59.8 V/cm range of electric field intensity at the centerline which was not optimal (Figure S2c). Upon closer inspection, a change in the applied voltage from 2 V to 3 V revealed that the presence of the porous hydrogel (triangles, electrical conductivity: σ = 2.98e‐8 S/m) had a larger effect in electric field changes than the electrolytic fluid alone (diamonds, electrical conductivity: σ = 0.346 S/m) (Figure S2d). These simulations helped guide the optimal range of applied voltage to achieve the desired electric field intensity at the hydrogel chamber.

### 2.4 YA‐ and OS‐derived myobundles differ in contraction rate and displacement signal

Human 2D and 3D cultured myotubes have been stimulated by chronic, low frequency electrical stimulation (1–2 Hz and 2 ms) to mimic physical exercise resulting in visible contractions and de novo formation of sarcomere structures (Khodabukus et al., 2019; Nikolić et al., 2017; Tarum, Folkesson, Atherton, & Kadi, 2017). Based on published results and our simulation studies, we tested electrical stimulation parameters on the YA myobundles as the healthy control using pulse regimes varying frequency from 1–3 Hz, pulse width by 0.5 or 2 ms, and voltage by 2–3 V. The myobundles attached to the microposts undergo isometric contraction. A periodic signal of contractile twitch response with clear tension and relaxation peaks of the muscle bundle was observed in YA‐derived chips when 3 V, 2 Hz, 2 ms parameters were applied at the parallel electrodes. The same stimulation parameters were selected for both YA‐ and OS‐derived myobundles because our objective was to observe differences in contractile response of the OS‐derived myobundles compared with the healthy controls (YA‐derived myobundles) and not to confound their responses to differences in stimuli.

**FIGURE 2:**
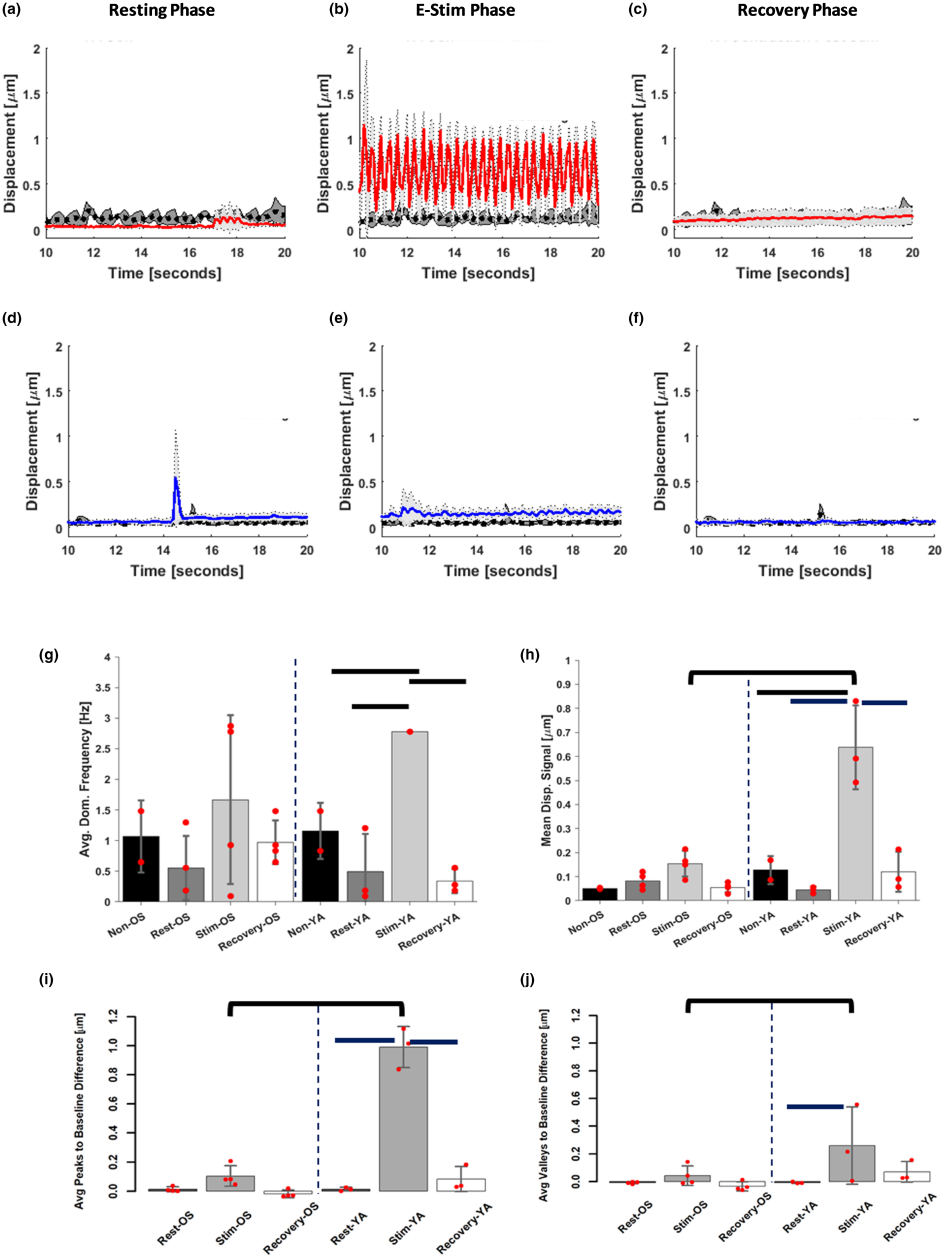
Contraction measurements of skeletal myobundles. (a–f) Displacement measurements were obtained using a digital image correlation algorithm on videos taken of (a–c) the YA (red lines) and (d–f) OS (blue lines) during resting phase (a, d), E‐Stim phase after applying a 3 V, 2 Hz, 2 ms electrical stimulation (b, e) and recovery phase (c, f) and compared with YA‐ and OS‐derived myobundles that did not undergo electrical stimulation (black dotted lines). Standard deviations are shown in light and dark gray shading on each graph for stimulated and non‐stimulated chips, respectively. (g–j) A Fast Fourier Transform analysis and standard signal processing techniques were applied to the displacement measurements to extract the average dominant frequency (g), mean displacement signal (h), average difference between displacement peaks and the sample resting mean displacement signal (i) and average difference between valleys or local minima and the sample resting mean signal (j). Bars, *p* < 0.05 between stimulation phases of cells within the same age group, and brackets between cells age groups at the same phase

We first differentiated the tissue chips for 13 days followed by an additional 5 days of differentiation in the presence of an applied electrical stimulation for a total of 17 days of culture. During the 5 days of electrical stimulation, we applied the 3 V, 2 Hz, with 2 ms pulse width sequence to the tissue chips for 30 min each day to mimic daily physical activity. Skeletal 3D myobundles have been reported to be stimulated for a maximum of 60 min with 7–12 h rest in between stimulation sequences (Khodabukus et al., 2019). A control group of tissue chips from each cohort did not receive electrical stimulation over the 5 days. At the end of the experiment on Day 17, image sequences of all tissue chips were recorded for a total of 120 s divided into the following phases: 40 s before (resting phase), during (E‐stim phase), and after (recovery phase) an applied electrical stimulation. Digital image correlation (DIC) analysis of these image sequences as described in Figure S3a–e provided an average myobundle displacement signal. The resultant displacement signals for each phase for YA‐derived tissue chips and OS‐derived tissue chips are shown in Figure 2a–c (YA) and 2d–f (OS). We display a portion of the timeframe (10–20 s) as a repeatable pattern. Analysis for the full 40 s recording is provided in the supplemental material. The YA‐derived tissue chips showed significantly higher magnitude of displacement and synchronous contraction rate in response to electrical stimulation compared with their resting phase baseline or to tissue chips that did not receive 5 days of electrical stimulation (Figure 2a, b and Video S1), whereas tissue chips formed from OS cells showed a non‐uniform waveform in response to electrical stimulation with a displacement magnitude close to their resting phase baseline (Figure 2d, e and Video S2). Average displacements measured at the resting and recovery phases for both YA‐and OS‐derived tissue chips followed limited or undefined patterns of contraction, as evidenced from the contraction peaks randomly observed over time (Figure 2a, c (YA) d, f (OS)). Tissue chips of YA cells that did not receive any stimulation also showed signs of spontaneous contractions with greater incidence than the non‐stimulated tissue chips derived from OS cells (Figure 2 black dotted lines).

Spectral analysis of the average displacement signals acquired between 10 and 20 s from the processed videos after 5 days of daily stimulation revealed a dominant frequency for YA‐derived tissue chips in response to electrical stimulation of 2.8 ± 0 Hz. We determined that our custom pulse generator delivered 2.8 Hz instead of 2.0 Hz. The minimum stimulation frequency necessary to evoke tetanus is 10 Hz; thus, our calculated frequency of 2.8 Hz is well within twitch parameters (Watanabe, Fukuhara, Fujinaga, & Oka, 2017).

The dominate frequency for the YA‐derived chips was significantly higher than the 0.5 ± 0.6 Hz and the 1.2 ± 0.5 Hz for YA‐derived tissue chips in their resting and non‐stimulated states, respectively (Figure 2g). Despite OS‐derived chips showing 94% coefficient of variation in contraction rate during their resting phase, they yielded significantly different frequencies during electrical stimulation than the spontaneous contractions observed during resting just like the case of YA‐derived tissue chips. DIC analysis for the full 40 sec recording are shown in Figures S4, S5.

The local maxima and minima of the mean displacement signals shown in Figure 2a–c, respectively, indicated the magnitude of the displacement during contraction and the extent of relaxation magnitudes experienced by the myobundles relative to the mean signal during their resting phase. With a mean displacement signal of 0.64 ± 0.17 μm, chips incorporating YA‐derived cells showed a 14.2‐ and 5.3‐fold increased contraction under electrical stimulation compared with their resting and recovery phases, respectively, and at least five times the displacement magnitudes of their non‐stimulated YA‐derived chips (Figure 2h). Conversely to the YA‐derived myobundles, the average displacement of stimulated tissues seeded with OS cells had negligible differences compared with their resting and recovery phases except for spontaneous contraction spikes.

The mean difference of contraction peaks to resting baseline produced by YA‐derived bundles under electrical stimulation was approximately 9.5 times greater than the difference in contraction peaks recorded from OS‐derived chips (Figure 2i, brackets). Analysis of local maxima and minima of the mean displacement signal obtained from each chip also indicated an average peak difference to resting baseline of 1.0 ± 0.1 μm for the YA‐derived chips under electrical stimulation and average difference of valleys to resting baseline (relaxation) of 0.3 ± 0.3 μm (Figure 2i, j). These average peak differences were significantly different than those of the same YA‐derived chips during their resting and recovery phases; however, only average valleys between stimulated YA‐derived chips and their resting counterparts were significant. No significant differences in mean displacement, normalized peak contractions, or valleys were detected among the OS‐derived test groups. A summary of the contraction parameters extracted from the DIC analyses for the 10–20 s and 0–40 s recordings are shown in Figure S5.

### 2.5 Sarcomere α‐actinin striation differs between YA‐ and OS‐derived myobundles

**FIGURE 3:**
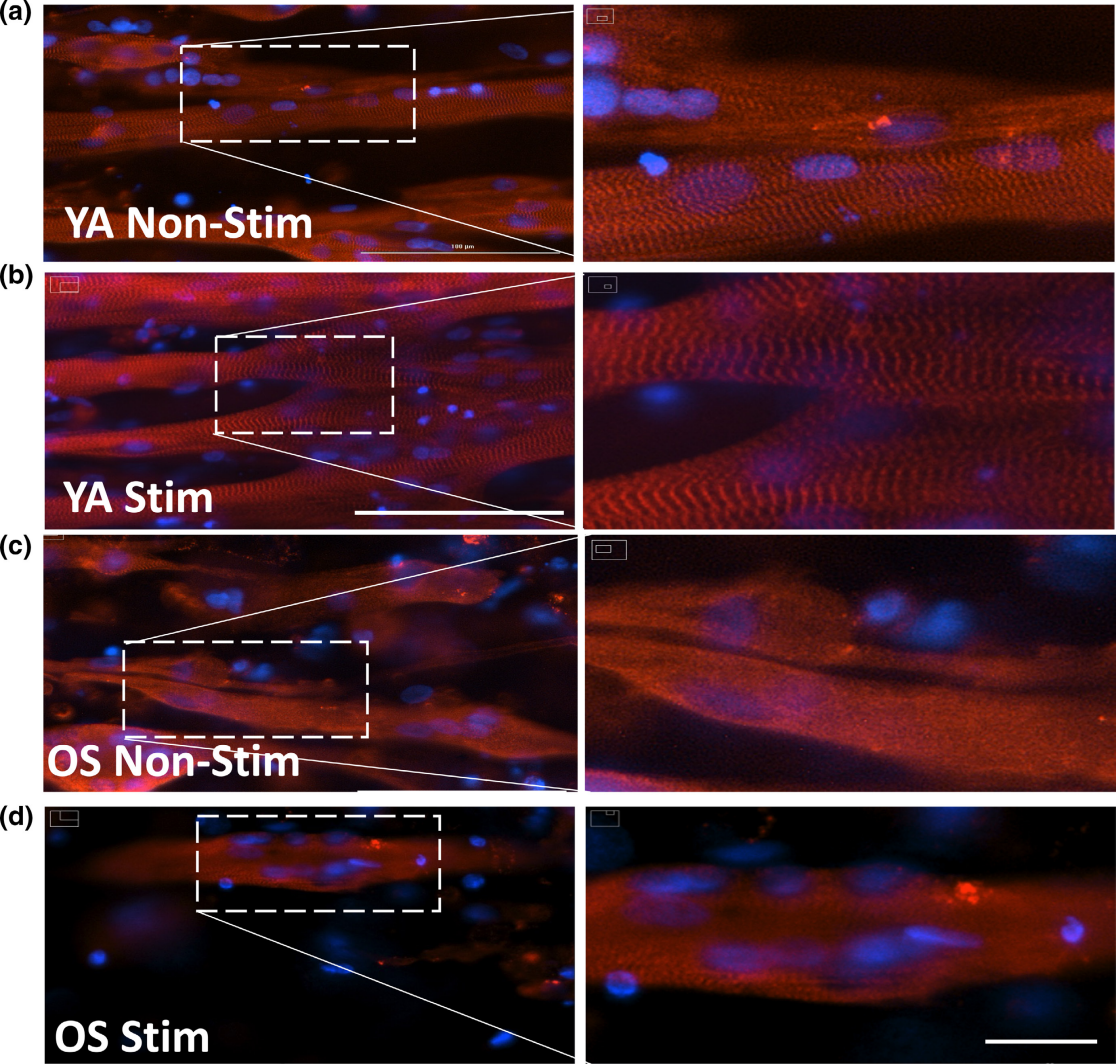
Characterization of 3D myobundles sarcomere by α‐actinin. YA‐derived myobundles non‐electrically stimulated for 5 days (a) and electrically stimulated (b) or OS‐derived myobundles non‐electrically stimulated for 5 days (c) and electrically stimulated (d) were stained with an anti‐sarcomeric α‐actinin antibody (red) and counterstained with the Dapi nuclear marker (blue) revealing the presence or absence of striated structure. Scale bars = 100 μm (a–d) and 20 μm (enlarged region of interest shown at right side in each figure shows cross‐striated appearance of skeletal muscle myobundles)

The significant difference in contraction displacement response between the YA‐ and OS‐derived electrically stimulated myobundles prompted us to investigate the expression of sarcomere α‐actinin contractile protein by immunofluorescence. Phenotypic analysis of protein staining showed that after differentiation, myoblasts formed multinucleated myotubes with diffuse striations in myobundles derived from the OS cohort that received electrical stimulation compared with the absence of striations in the myobundles derived from the OS cohort that did not receive electrical stimulation (Figure 3c, d). Myotube thickness appeared similar. This contrasts with extensive striations observed in myobundles derived from the younger cohort whether the chips were electrically stimulated or not (Figure 3a, b). The difference in the YA‐derived myobundles that received electrical stimulation was increased fusion of myotubes and thus, increase in myotube size compared with myobundles that did not receive electrical stimulation. The OS‐derived myobundles appeared to be shorter compared with myobundles from the younger cohort.

### 2.6 Myogenesis and contractility genes are upregulated in skeletal muscle myobundles

**FIGURE 4:**
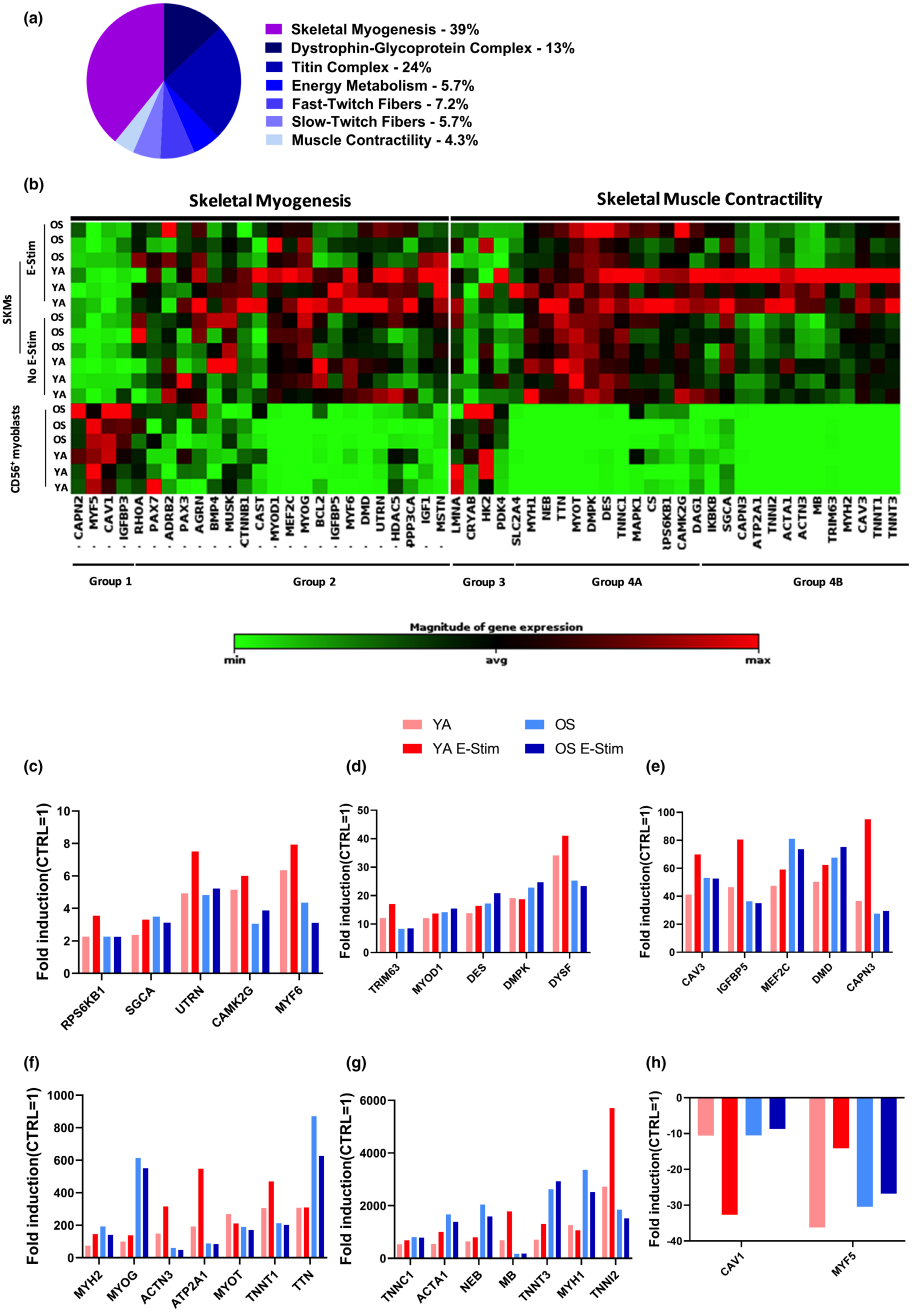
Gene expression profiling of YA‐ and OS‐derived skeletal muscle myobundles. (a) Myogenesis and Myopathy RT2 Profiler PCR Array gene categories and percentages. (b) Clustergram indicates non‐supervised hierarchical clustering of 55 genes involved in skeletal myogenesis and skeletal muscle contractility in undifferentiated myoblasts and differentiated skeletal muscle cells (SKMs) with (E‐Stim) or without (no E‐Stim) applied electrical stimulation. X‐axis indicates genes with similar expression profiles. Y‐axis indicates sample similarity. Genes with high, average, or no expression levels are depicted in red, black, and green, respectively (see color bar). (c–h) Fold induction (FC ≥2, *p*‐value ≤0.05) of shared upregulated (c–g) or downregulated (h) genes with significant differential expression in differentiated SKMs with or without E‐Stim normalized to YA and OS undifferentiated CD56^+^ myoblast values. Data are representative of three independent RT2 Profiler PCR array determinations (*n* = 3 tissue chips for each cohort tested in duplicate)

Following the differentiation and electrical stimulation protocol, skeletal myobundles were preserved for RNA extraction and RT‐PCR analysis. We compared the expression levels between the YA and OS cohorts of 55 genes implicated in skeletal myogenesis and skeletal muscle contractility signaling pathways (Figure 4a). RNA isolated after 2 or 16 days of differentiation with (E‐Stim) or without (No E‐Stim) applied electrical stimulation are displayed by clustering the log2 fold change for each gene in a heatmap‐dendrogram (Figure 4b). Major differences in expression patterns occurred between undifferentiated myoblasts and differentiated myobundles regardless of whether the myobundles received applied electrical stimulation. Based on the patterns of expression, four main groups of genes were identified. Group 1 myogenesis‐related genes in both cohorts were upregulated in the myoblasts and switched to being downregulated or unchanged after differentiation indicating early biomarker genes such as MYF5 and CAV1 are dialed down during myotube development. Group 2 myogenesis‐related genes and group 4 A/B contractility‐related genes were downregulated in both YA‐ and OS‐derived myoblasts and switched to being significantly upregulated by varying degrees after differentiation highlighting functional maturation of the cells regardless of cell phenotype. Finally, Group 3 contractility‐related genes in the myoblasts from YA cohorts had increased expression levels. Interestingly, gene expression changes displayed in the heatmap‐dendrogram appear different between the E‐Stim and non‐Stim tissue chips regardless of cell phenotype.

We set a threshold for differentially expressed genes (DEGs) compared with the expression of genes in myoblasts after 2 days of growth of fold change >2 and *p* < 0.05. Overall, 34 and 33 of the 55 genes investigated were DEG and upregulated in OS‐ and YA‐derived myobundles, respectively. Only a single gene and 4 genes were downregulated DEGs in YA‐ and OS‐derived myobundles, respectively, after differentiation as illustrated in the volcano plots (Figure S6a, b). The complete list of genes, fold‐change, and p‐values for each treatment group are shown in Figure S7. Twenty‐nine of the DEGs upregulated and 2 downregulated were shared across all four categories (YA no E‐Stim, YA E‐Stim, OS no E‐Stim, and OS E‐Stim) as depicted in the Venn diagrams (Figure S8a, b). The 29 up‐regulated DEGs in both differentiated YA and OS with or without E‐Stim were processed using the STRING v10 WEB based bioinformatics tool (Szklarczyk et al., 2011) which generated protein interaction network with three clusters using highest confidence interaction scores (≥0.9) and hiding disconnected nodes from the network (Figure S9). The 29 DEGs were annotated according to the STRING database and the top 5 gene ontology (GO) terms based on the false discovery rate scores of biological processes, molecular functions, and cellular components, as summarized in Figure S10.

The fold induction of these shared DEGs in each cohort with and without E‐Stim are depicted in increasing order in the bar charts (Figure 4c‐h). The YA E‐Stim myobundles displayed a trend toward higher levels of myogenesis genes including utrophin (UTRN), myogenic factor 6 (MYF6), and myosin heavy chain 2 (MYH2). Expression of genes involved in myofibril assembly was also significantly upregulated during differentiation such as α‐sarcoglycan (SGCA), caveolin 3 (CAV3), dysferlin (DYSF), and calpain 3 (CAPN3). Furthermore, genes annotated to increase fiber composition and confer calcium‐sensitivity to skeletal muscle such as α‐actinin‐3 (ACTN3), Duchenne muscular dystrophy (DMD), nebulin (NEB), slow skeletal muscle troponin T1 and T3 (TNNT1/3), troponin I2 (TNNI2), troponin C1 (TNNC1), and titin (TTN) were also significantly upregulated in both young and old derived myobundles.

**FIGURE 5:**
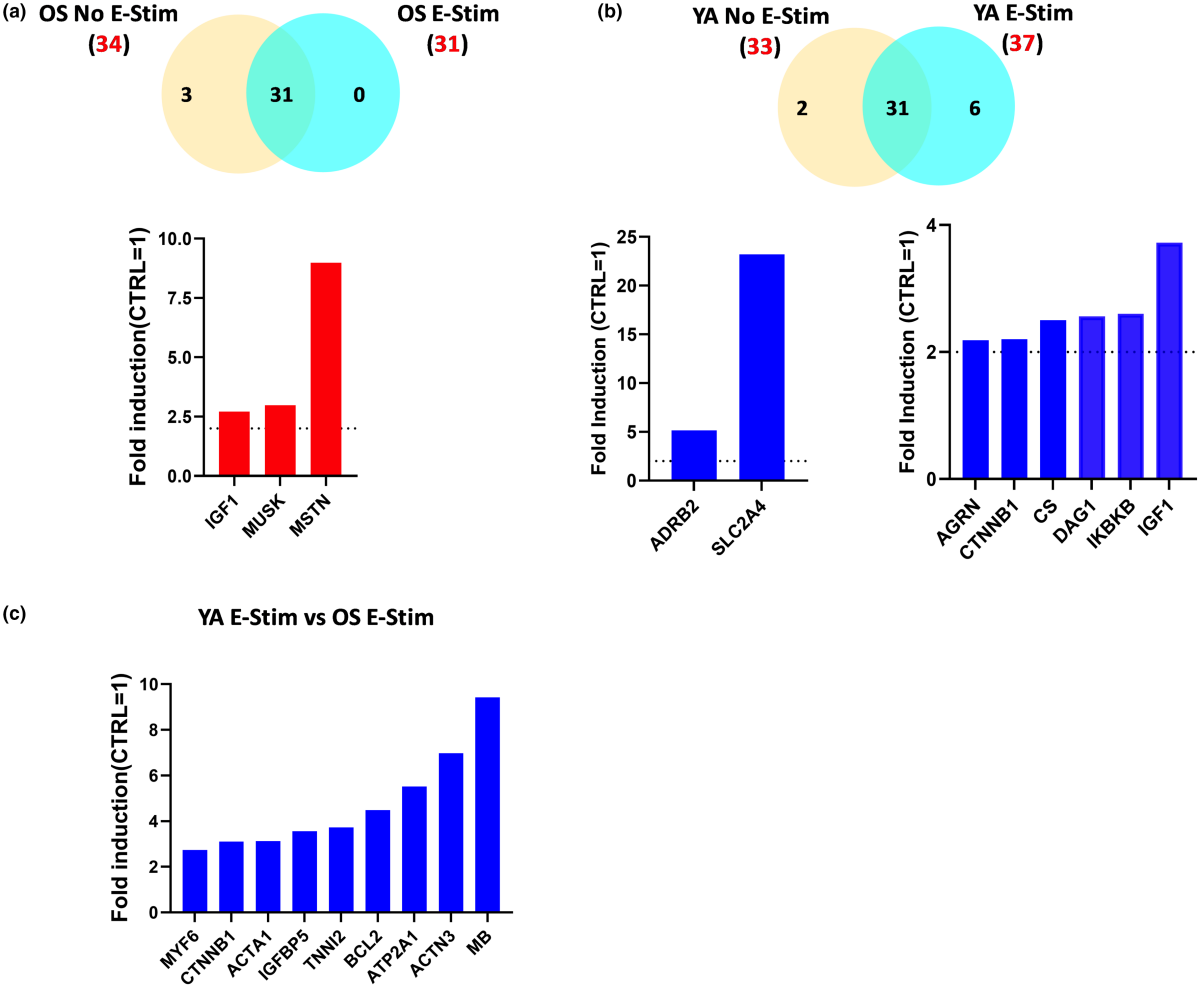
Gene expression signature of YA and OS skeletal muscle bundles cohorts with or without E‐Stim. (a) Venn diagram representing statistically significant up‐regulated genes from differentiated OS no E‐Stim vs. undifferentiated OS CD56^+^ satellite cells (yellow, 34 genes) and from differentiated OS E‐Stim vs. undifferentiated OS CD56^+^ satellite cells (blue, 31 genes) showing an overlap of 31 genes of interest between the two sets of samples (up). Bar charts depicting statistically significant genes upregulated only in differentiated OS no E‐Stim (bottom). (b) The Venn diagram depicts statistically significant up‐regulated genes from differentiated YA no E‐Stim vs. undifferentiated YA CD56^+^ satellite cells (yellow, 33 genes) and from differentiated YA E‐Stim vs. undifferentiated YA CD56^+^ satellite cells (blue, 37 genes) showing an overlap of 31 genes of interest between the two sets of samples (up). Bar charts depicting statistically significant genes up‐regulated only in differentiated YA no E‐Stim (left) and only in differentiated YA E‐Stim (right) and normalized to undifferentiated YA CD56^+^ myoblast cells. (c) Bar chart depicts statistically significant genes up‐regulated only in differentiated YA E‐Stim when compared to differentiated OS E‐Stim. Data are representative of three independent RT2 Profiler PCR Array determinations (*n* = 3 tissue chips for each cohort tested in duplicate)

To confirm gene responses specifically induced with electrical stimulation, we cross‐compared groups. Three DEGs including insulin growth factor‐1 (IGF‐1), muscle associated receptor tyrosine kinase (MUSK), and myostatin (MSTN) were expressed in the OS no E‐Stim but not in the OS E‐Stim group and no unique DEGs were observed in the OS E‐Stim compared with OS no E‐Stim group (Figure 5a). Two DEGS including adrenoceptor beta 2 (ADRB2) and Solute Carrier Family 2 Member 4 (SLC2A4) were expressed in the YA no E‐Stim but not in the OS E‐Stim group and 6 unique DEGs were observed in the YA E‐Stim compared with YA no E‐Stim group (Figure 5b). Next, we examined the muscle tissue response to electrochemical stimulation by comparing differentiated YA E‐Stim versus OS E‐Stim. We found 9 up‐regulated DEG in YA E‐Stim‐ vs. OS‐Stim‐myobundles known to be involved in muscle cell differentiation and contraction (Figure 5c).

## 3 DISCUSSION

We describe the development of an electrode embedded microfluidic tissue chip to study phenotypic and physiological differences between human skeletal muscle myobundles generated from pooled primary cells as a model to study age‐related muscle atrophy. We provide a comprehensive analysis of the difference in contraction displacement rates, gene expression profiling, and immunostaining, and validate this model to be used as ground studies for spaceflight and for therapeutic discovery. It should be kept in mind that for this study, the reported differences may not be related to just an age difference, but to a different level of muscle activity as the satellite cells were from active young individuals and sedentary old individuals. Future studies will be necessary to determine if this approach can be used to determine differences between sedentary vs. active individuals, whether they are young or old by studying cells from young sedentary, old active participants, or older patients with muscle pathology to further refine the in vitro model of aging and age‐related disease.

The multinucleated, aligned myobundles recapitulate key features of native tissue and exhibit robust myofiber differentiation due to high cell density, relevant collagen‐Matrigel hydrogel, and dynamic culture conditions. The combination of these features and the formation of a single myobundle induces spontaneous and robust contractile performance in response to low frequency electrical stimulation in the YA‐derived healthy myobundles. Importantly, contraction is a characteristic of muscle that underlies key features of muscle phenotype in vivo that are not fully recapitulated in 2D models of skeletal muscle (Eberli, Soker, Atala, & Yoo, 2009; Guo et al., 2014).

Although the OS‐derived myobundles exhibit the same 3D architecture as their YA counterparts, these aged myobundles induce residual, non‐synchronic contraction in response to an acute electrical stimulation regime. The differences we observe in muscle contractile characteristics between the YA‐ and OS‐derived myobundles are in line with reports that donor age can impact other key phenotypes that are retained in primary myotubes, including substrate oxidation (Aas et al., 2020). Thus, our MPS provides a physiologically relevant platform to study mechanisms involving molecular and functional response differences in aging muscle.

We profiled the expression of 55 genes relating to myogenesis and myopathy, a majority of which were upregulated similarly in YA‐ and OS‐derived myobundles during the two‐step differentiation process compared with their respective myoblast stage. Key transcriptional activators that promote transcription of muscle‐specific target genes involved in muscle cell fate commitment were also upregulated including myoblast determination 1 (MYOD1, 10‐fold), myogenic factor 6 (MYF6, 2‐3‐fold), myogenin (MYOG, 100‐600‐fold), and myocyte enhancer factor 2C (MEF2C, 40‐80‐fold). Paired box 7 (PAX7) has no expression and myogenic factor 5 (MYF5, −10‐35‐fold) is significantly downregulated in all groups. These trends mimic adult muscle under normal conditions where PAX7 and MYF5 are active in quiescent satellite cells and decrease during myoblast activation, MYOD1 increases during myoblast activation and MYOG and MRF6 are upregulated during muscle differentiation and formation of myotubes (Berkes & Tapscott, 2005; Vepkhvadze, Vorotnikov, & Popov, 2021; Zammit et al., 2004). These data indicate that primary muscle cells cultured in the MPS can proliferate and differentiate as expected.

We electrically stimulated the mature myobundles with a 3 V, 2 Hz, 2 ms pulse sequence through platinum electrodes aligned in the media channel. Electrochemical impedance spectroscopy conducted on our MPS geometry allowed us to inform theoretical simulations of electric currents using COMSOL Multiphysics to identify appropriate electric field magnitudes and current distribution across the chip. Our evaluations indicate that 3 V applied at the parallel electrodes spaced 0.8 cm apart produced a homogeneous distribution of an electric field across the tissue. When accounting for the presence of the hydrogel and the media conductivity, our predicted stimulation intensity was within the range of published recordings in bioengineered skeletal muscle systems generated from both primary and IPSC‐derived human skeletal muscle sources (Madden et al., 2015; Rao, Qian, Khodabukus, Ribar, & Bursac, 2018; Shima, Morimoto, Sweeney, & Takeuchi, 2018; Shimizu et al., 2015; Takahashi, Shimizu, & Okano, 2018).

Electrical stimulation robustly induced the expression of skeletal muscle specific cytoskeleton components central to the contractile machinery such as α ‐actin 1 (ACTA1, 500‐ and 1500‐fold), α‐actin 3 (ACTN3, 300‐ and 50‐fold), myosin heavy chain 2 (MYH2, 50‐ and 200‐fold), and myosin heavy chain 1 (MYH1, 1000‐ and 3000‐fold) between YA‐ and OS‐derived myobundles, respectively. Titin and associated proteins are also important components of the myofilament system (Granzier & Labeit, 2005). In the YA‐derived myobundles, titin (TTN), troponin T1 (TNNT1), troponin I2 (TNNI2), and T3 (TNNT3) genes are upregulated by 500‐, 300‐, 2500‐, and 600‐ fold, respectively. With electrical stimulation, genes increased in expression by an additional 1.5‐, 2‐, and 2‐fold for TNNT1, TNNI2, and TNNT3, respectively. These genes highlight the myofiber mix between fast and slow twitch muscle type characteristic of vastus lateralis and induced by electrical pulse stimulation in cultured human myotubes (Marš et al., 2021; Staron et al., 2000). Alpha‐actinin immunofluorescence staining of the YA‐derived myobundles confirmed well‐defined z‐line architecture in the fused myotubes. No genes were specifically induced by electrical stimulation in the OS‐derived myobundles. Nonetheless, non‐stimulated OS‐derived myobundles such as non‐stimulated YA‐derived myobundles exhibited 3–4 orders of magnitude upregulation of titin‐troponin family of genes; TTN, TNNT1, TNNI2, and TNNT3 were upregulated by 800‐, 1800‐, 200‐ and 3000‐fold, respectively, in OS‐derived myobundles. Despite, the high expression levels for actin, myosin, and titin‐related genes, α‐actinin immunofluorescence staining of the OS‐derived myobundles exhibited diffuse striations after 5 days of electrical stimulation.

In addition, in our model, insulin growth factor 1 (IGF‐1) and myostatin, the negative regulator of muscle growth, were induced upon electrical stimulation in the YA‐derived myobundles but attenuated in electrically stimulated OS‐derived myobundles. In addition to decreased muscle growth compared with younger individuals, aging is associated with a variable hypertrophic response after acute exercise training (Welle, Totterman, & Thornton, 1996). Under normal conditions, gene expression analysis determined in muscle of young and old subjects indicated that both IGF‐1 and myostatin are attenuated in adults >80 years old (Naro et al., 2019). Our results highlight the variable hypertrophic response and the balance between pro‐ and anti‐atrophy gene expression in the older cohort under an acute electrical stimulation regime.

An advantage of our contraction analysis is that we determine a displacement signal over time from the average displacement magnitude of the bundle in the horizontal and vertical direction across a sizable region of interest (described in Figure S2). Our results indicate that the YA‐derived myobundles showed mean displacement magnitude of contraction 4× the mean displacement value of OS‐derived myobundles (Figures 2h and S5). Our analysis also detected regions of the myobundle that moved in a preferentially axial orientation and others which condensed inward with contraction. The regions of large displacement magnitudes consisted of the boundaries where the myobundle had stretched away from the PDMS posts and the outer edges where the cells had condensed away from the microfluidic chamber. Interestingly, these same regions, which may have greater oxygen transport (Truskey, 2018), showed high intensity of immunofluorescent staining of myosin and α‐actinin proteins, suggesting that greater contractions are associated to locations where myotube differentiation and sarcomere structures are most prominent (Wang et al., 2021). We anticipate that incorporation of more frequent electrical stimulation pulses over many more days and response to compounds may change this displacement pattern.

By evaluating the vector sum of the horizontal and vertical displacements across the myobundle region, we also determined the mean displacement signal with periodic contraction peaks and valleys for each chip. The upward or downward trends of the mean signal indicated whether the myobundles remained under increasing tension or if they were relaxing during the corresponding stimulation phase. The mean displacement signal and average difference of peaks and valleys with respect to the resting baseline, indicated that YA‐derived myobundles responded to the selected stimulation parameters with 1 μm peak displacement and returned to baseline (Figures 2i, j and S5) to undergo greater depolarization of the cell membrane leading to muscle cell contraction compared with OS‐derived myobundles which showed negligible peak displacement. In addition, the YA‐derived myobundles contracted on average 5–8 times faster than during their resting or recovery phases. The synchronous signal of YA‐derived tissue chips under electrical stimulation followed by a period of negligible frequency of low intensity contractions in the recovery phase indicates that the YA‐derived myobundles undergo relaxation after electrical stimulation is removed. In OS‐derived myobundles, a higher frequency twitch pattern of low intensity contractions was detected compared with the resting phase, suggesting that OS‐derived myobundles may possess insufficient myotube density or sarcomere structures to generate a synchronous contraction response. Our immunofluorescence sarcomere staining supports this hypothesis.

Native muscle contraction is a three‐dimensional phenomenon, and this nature influences its mechanical output and is dependent on variations in muscle shape change (Roberts et al., 2019). Our in vitro system that mimics the multi‐scale nature of muscle mechanics can be enhanced with different electrical stimulation “exercise” regimes and may provide a link between structure and functional changes that occur during aging. Furthermore, by studying the physiological effects of myobundles derived from myoblasts from participants phenotyped based on age and physical activity and comparing effects on muscle biology in microgravity, our data stand to highlight the distinct impact that aging and physical activity combined have on human muscle energetics.

## 4 MATERIALS AND METHODS

### 4.1 Chemicals

Dow SYLGARD™ 184 Silicone Elastomer Clear was purchased from Ellsworth adhesives (Germantown, WI). Platinum wire was obtained from Surepure Chemetals (Florham Park, NJ). (2*S*)‐*N*‐(Watanabe et al., 2017)‐L‐alanyl‐2‐phenyl]glycine 1,1‐dimethylethyl ester (DAPT) was obtained from Tocris chemicals (Minneapolis MN). Dabrafenib was ordered from APExBIO (Boston, MA). Premix supplement of insulin (5 μg/mL), transferrin (5 μg/mL), and selenious acid (5 ng/mL) (ITS) was obtained from Corning (Corning, New York). Gibco B‐27 supplement was from ThermoFisher (Waltham, MA), and primocin was ordered from InvivoGen (San Diego, CA). Sterile dimethyl sulfoxane (DMSO) was purchased from Sigma‐Aldrich (St. Louis, MO). Paraformaldehyde (PFA) was purchased from Electron Microscopy Sciences (Hartfield, PA). Unless otherwise indicated, materials listed below were obtained from ThermoFisher (Waltham, MA).

### 4.2 Design and chip fabrication

Chip designs were created using Solidworks 2020 (MLC CAD Systems, LLC, Austin, TX). Chip molds were 3D printed by Accura 60 High‐Resolution Stereolithography build in 0.002″ layers (Proto Labs, INC, Maple Plain, MN). Polydimethylsiloxane (PDMS) chips were prepared by mixing elastomer base and encapsulant (10:1) and pouring into the 3D platinum‐containing molds and placing under vacuum for 15 min to remove air. Platinum wires (24 mm) were secured into the molds, and the molds were cured at 50°C for 6 h followed by 1 h at ambient temperature. The cured PDMS was removed from mold and 1 mm biopsy punch was used to generate ports prior to bonding the PDMS to glass slides of various sizes by plasma cleaning PDMS and glass for 60 s under vacuum using a Harrick plasma cleaner. Bonded chips were sterilized with ethanol and heated at 150°C for 30 min prior to cell seeding.

### 4.3 Study design and participants

Volunteers were recruited to participate in this study from the Orlando, FL area. Volunteers were eligible to participate if they were weight stable (±4.5 kg in preceding 6 months), had a body mass index (BMI) between 20 and 35 kg/m^2^, and were in good general health. Volunteers were excluded if they were taking medications known to influence muscle metabolism, had a chronic medical condition (diabetes, cardiovascular disease, and cancer), had any contraindications to exercise, had high resting blood pressure (≤150 mmHg systolic, ≤90 mmHg diastolic). Screening and clinical phenotyping were completed over four study visits at the Translational Research Institute (TRI) at AdventHealth, Orlando. The screening visit consisted of a fasting blood draw, physical measurements, medical history/physical activity questionnaires, and resting electrocardiography (ECG). On Visit #2, participants completed a VO_2_ peak test with ECG to determine cardiorespiratory fitness (Distefano et al., 2018). On Visit #3, participants completed magnetic resonance imaging (MRI), and a dual energy X‐ray absorptiometry (DXA) scan to assess body composition and quadriceps contractile and physical function testing, as previously described (Distefano et al., 2018). All participants provided written informed consent, and the study protocol was reviewed and approved by the institutional review board at AdventHealth, Orlando (IRBNet #554559). Thus, the study was performed in accordance with the ethical standards laid down in the 1964 Declaration of Helsinki.

### 4.4 Percutaneous muscle biopsy and skeletal muscle cell isolation

On the final study visit, participants arrived in a fasting state. Participants then consumed a small low glycemic index meal (200 kcal, 15% protein, 35% fat, and 50% carbohydrate), and 15 min later, a skeletal muscle biopsy procedure was conducted. Participants were instructed not to perform physical exercise 48 h prior to the biopsy procedure. Biopsy samples were obtained from the middle region of the vastus lateralis under local anesthesia (2% buffered lidocaine) as described previously. A portion of the biopsy specimen was used for satellite cell isolation. Satellite cells (quiescent mononuclear muscle cells) were isolated by trypsin digestion, pre‐ plated on an uncoated petri dish for an hour to remove fibroblasts, and subsequently transferred to T‐25 collagen–coated flasks in Dulbecco's Minimum Essential Medium (DMEM, Gibco) supplemented with 16% fetal bovine serum (FBS, Gibco) and human growth factors. Cells from individual donors were passaged twice, trypisinized, centrifuged, and resuspended at 10 × 10^6^ cells/mL in 90% FBS and 10% DMSO, labeled with AdventHealth ID number stored at −170°C.

### 4.5 CD56 ^+^ cell enrichment

Pooled HSKM cells were thawed and cultured on collagen I (Corning, Corning, NY) coated T75 flasks in Skeletal Muscle Cell Growth medium (PromoCell, Heidelberg, Germany). When cells reached 70%–80% confluency, they were expanded by trypsinization for no more than two expansions. Mononuclear myoblasts were immunopurified using the mouse monoclonal 5.1H11 anti‐CD56 antibody (5.1H11 was deposited to the DSHB by Blau, H.M./Walsh, F.S. (DSHB Hybridoma Bank, Iowa City, IA) and CD56^+^ myoblasts were enriched using MACS magnetic bead technology (Miltenyi Biotec, Auburn, CA), according to the manufacturer's protocol. Viability of enriched cells was assessed using trypan blue exclusion. Enrichment was confirmed by comparing CD56^−^ and CD56^+^ cell populations by FACS staining as described in Supplemental Material (Figure S1).

### 4.6 Fabrication of myobundles and differentiation

Enriched myoblasts were carefully mixed with a hydrogel composed of 3.3 mg/mL rat tail collagen I and 22% (v/v) Matrigel1 for a final cell density of 15–20 × 106/mL. Acid‐solubilized rat tail collagen I (ibidi, Fitchburg, WI) was salt balanced using 10× Dulbecco's Phosphate Buffered Saline and pH neutralized using 1 N NaOH on ice prior to being mixed with Matrigel (Corning, Corning, NY). Myoblasts were resuspended directly into hydrogel and immediately seeded into tissue chips using a 1 mL syringe (BD Biosciences, San Jose, CA) fitted with a 20G blunt needle. The cell laden hydrogel was polymerized at 37°C for 30–60 min. Seeded tissue chips were connected to a syringe pump containing Skeletal Muscle Cell Growth Medium (PromoCell) supplemented with 0.1 mg/mL Primocin (InvivoGen, San Diego, CA) and fed for 2 days at a constant flow rate of 1 mL/h. After 2 days in growth media, cells condensed down to form a muscle bundle wrapped around the two PDMS posts. Media was switched to Differentiation Media 1 (MEM‐α, 0.5% (v/v) ITS, 2%(v/v) B27, 10 μM DAPT, 1 μM Dabrafenib, 20 mM HEPES, pH 7.3 and, 0.1 mg/mL Primocin) and pumped at an intermittent rate of 125 μL/min for 1 min every 8 h for 7 days. Day 7 differentiated bundles were switched to Differentiation Media 2 (MEM‐α, 0.5% (v/v) ITS, 2% (v/v) B‐27, 20 mM HEPES, pH 7.3 and 0.1 mg/mL Primocin) and pumped for an additional 7 days. All media used in tissue chips were degassed using house vacuum and a heated stir plate (37°C, 300 rpm).

### 4.7 Immunofluorescence staining of skeletal muscle myobundles

Differentiated muscle bundles were fixed and immunostained directly in the tissue chips. Bundles were thoroughly washed with 1× PBS and then fixed for 15 min at room temperature with 4% PFA. Fixed bundles were thoroughly washed with 1× PBS. Bundles were permeabilized and blocked using TrueBlack Background Suppressor (Biotium, Fremont, CA) for 10 min at room temperature. Bundles were incubated with either the myosin heavy chain antibody clone MF‐20 (MF 20 was deposited to the DSHB by Fischman, D.A. (DSHB Hybridoma Bank, Iowa City, IA)) or the sarcomeric α‐actinin antibody (clone EA‐3) prepared in TrueBlack Blocking Buffer (Biotium) overnight at 4°C. Bundles were washed by incubating in 1X PBS for 30 min at room temperature followed by a thorough flush with 1× PBS. The secondary antibody, goat anti‐mouse IgG (H + L)‐Texas Red, was prepared in TrueBlack Blocking Buffer and incubated with bundles for 2 hr at room temperature. Bundles were washed by incubating in 1× PBS for 30 min at room temperature followed by a thorough flush with 1× PBS. Nuclei were stained using 1:1000 DAPI and incubated at room temperature for 10 min. Background autofluorescence generated by the hydrogel was quenched by incubating the bundle with 1× TrueBlack® Lipofuscin Autofluorescence Quencher (Biotium) for 30 s. Bundles were thoroughly flushed with 1× PBS and stored in 1× PBS prior to imaging or preserved using VECTASHIELD vibrance antifade mounting media (Vector Laboratories).

### 4.8 Image analysis of myobundles

Immunofluorescence images were acquired using a Biotek Cytation5 Imager (Agilent). Myosin heavy chain images were obtained using a 20× objective and Sarcomeric α‐actinin images were obtained using a 40× objective. The Texas Red LED cube/filter pair was used in both cases to capture montage image tiles and Z slices. DAPI was used to stain nuclei. Image processing was performed using the Biotek Gen5 Image Prime software with Spot Counting Module (Agilent) to enhance the contrast between nuclei and fused myotubes. Nuclei were counted by creating a mask around individual nuclei (Total Nuclei). Fused myotubes were identified by creating a mask around all myosin heavy chain positive myotubes (Myotubes). Nuclei within fused myotubes were identified and counted using the Spot Counting module (Nuclei in Myotubes). Settings for spot counting were adjusted to create a spot mask on nuclei present with myotubes. Fusion Index was calculated as (Nuclei in Myotubes/Total Nuclei)*100. Myotube alignment orientation angles were measured using ImageJ's Angle Tool. Each myotube angle was measured from the mean orientation axis, the straight line drawn between PDMS posts. Each measured myotube angle was grouped in increments of 10°. Each angle group was given an alignment score calculated as (# of measured myotubes in angle group/total measured myotubes)*100.

### 4.9 Electrical field simulation in tissue chip

While the electric field cannot be measured directly, the electric potential difference or voltage change between two points can be obtained from direct measurements. In the case of the tissue chip, the electrodes exposed to conductive media formed an electrochemical cell at the interface between the liquid electrolyte‐rich media and the solid metallic electrodes. An electrochemical impedance spectroscopy (EIS) technique commonly used to evaluate commercial batteries (Wang et al., 2021) was used to evaluate the transient response of the tissue chip system and identify the ohmic resistance of the differentiation media. The conductivity of differentiation media was calculated from the ohmic resistance obtained by applying a 10 mV at a frequency sweep from 0.1 to 10^5^ Hz and measuring the alternating current (AC) response in a 2‐point configuration with a 1040C multi‐potentiostat (CHI Instruments, Inc.). With the known conductivity values and the known voltage applied at the electrodes in the tissue chip, finite element models were developed in COMSOL Multiphysics (Burlington, MA) to calculate the electric field intensity and distribution of currents within the chip. The electric currents (ec) interface in the basic COMSOL package with an added Microelectromechanical Systems (MEMS) module was used to simulate direct current (DC) stimulation from an electric potential applied between electrodes after the chip had been flooded with sodium ions diluted in differentiation medium. The distribution of sodium ions throughout the media and within the hydrogel was obtained from previously implemented flow simulations using the free and porous media flow (fp) and the transport of diluted species interfaces (tds).

### 4.10 Electrical stimulation of engineered 3D myobundles

3D myobundles were electrically stimulated daily for 30 min using a custom control system based on a programmable intelligent computer (PIC) microcontroller (microchip.com) and a custom electronics circuit board to receive input control signals from a graphical user interface (GUI) Windows‐laptop customer software program. The GUI program was developed using Microsoft Visual Studio. The GUI software communicates with the E‐Stim control system through a USB serial interface. The GUI sends out user‐selected control commands to set stimulation pulse duration, pulse frequency, and pulse voltage levels which are customizable. On the E‐Stim system, the microcontroller then uses a Digital to Analog Converter (DAC) to set the amplitude level. Pulse frequency and duration are set by commanding the DAC on and off by the microcontroller. Pulse frequency was validated by an Owon Digital Storage Oscilloscope with VGA Port, 2 Channels, 30 MHz, 250 MS/s Sample Rate. The microcontroller firmware was custom developed using American National Standards Institute for the programming C language (ANSI‐C).

### 4.11 Myobundle displacement and contraction rate determination

On Day 17 of differentiation and after five consecutive days of electrical stimulation, the tissue chips were placed in an environmentally controlled Cytation 5 high content imager (Biotek, Winooski, VT) and reconnected to the pulse generator described above. Images of the 3D myobundles were captured at 10 frames per second using a 20× objective. Three 40‐s image sequences were obtained for each chip: (1) before electrical stimulation (resting), (2) during electrical stimulation (E‐Stim), and (3) after stimulation was turned off (recovery). An open‐source digital image correlation (DIC) algorithm (Ncorr v.1.2) (Blaber, Adair, & Antoniou, 2015) based in MATLAB (Mathworks, Natick, MA) was used to quantify displacements at the region of interest encompassing the myobundle between the PDMS pillar and the tissue edge as previously implemented (Mojica‐Santiago et al., 2018). From the axial (horizontal) and vertical displacement maps obtained via DIC, magnitudes of the resultant displacement vectors in the two‐dimensional ROI were computed. To discretize the contraction within the region of interest of each chip into a one‐dimensional time‐dependent signal, the average displacement of the nonzero magnitudes in the entire ROI excluding the background was reported at each timepoint. The periodic behavior of the resultant signal was subsequently analyzed using conventional spectral analysis techniques in R‐Studio. A Fast Fourier Transform of the time series signal of displacement was used to obtain the dominant frequency of contraction in each chip. Further analysis of local maxima and minima was applied to evaluate differences in tissue contraction by experimental conditions.

### 4.12 Gene Expression Array studies

RT2 Profiler PCR pathway focused arrays were applied to detect changes in gene expression of skeletal muscle growth and differentiation. Total RNA from human CD56^+^ enriched cells with or without electrical stimulation for 5 days were isolated with RNeasy Plus Mini Kit (Qiagen, Germantown, MD) according to the manufacturer's instructions. 400 ng of total RNA from tissue chips were converted by Reverse transcription according to the manufacturer's instructions (Qiagen, Germantown, MD) and the resulting cDNA were frozen at −20°C. The cDNA was subjected to the Human Skeletal Muscle: Myogenesis & Myopathy (PAHS‐099ZE) PCR arrays. 10 μL PCR components mix were added to each well of the RT2 Profiler PCR Array and amplified as follows: 10 min of initial denaturation at 95°C, 40 cycles at 95°C for 15 s, 60°C for 1 min for annealing, and one additional step for melting curve 15 s at 95°C and 60°C for 15 s by using 12 Flex QuantStudio (Applied Biosystems, Foster City, CA). The results of each RT2 Profiler PCR Plate were subjected to data quality control checks (using the GeneGlobe Program, for monitoring genomic DNA contamination (GDC), the first strand synthesis (RTC) as well as real‐time PCR efficiency (PPC) following the manufacturer's instructions. Each RT2 Profiler PCR Array contains five housekeeping genes (ACTB, actin beta; B2M, β‐2 microglobulin; GAPDH, glyceraldehyde 3‐phosphate dehydrogenase; HPRT1, hypoxanthine phosphoribosyltransferase 1; and RPLP0, ribosomal protein lateral stalk subunit P0). The geometric mean of the CT values for the five housekeeping genes was calculated and subtracted from the CT value of each gene of interest to obtain ΔCT values (Vandesompele et al., 2002).

### 4.13 Statistical analysis

All experimental measurements report error as standard deviation and the statistical significance between test groups (i.e., # YA chip replicates vs. OS chip replicates) was determined by paired *t*‐tests. For clinical data presented in Table 1, group differences were determined using a non‐paired students *t*‐test. The statistical significance of the differences in contraction parameters between the OS‐ and YA‐derived chips at the different stimulation phases was determined using a linear mixed‐effects model in R‐Studio (Bates, Mächler, Bolker, & Walker, 2015). The stimulation phase, cell type, and interactions were considered as fixed effects, while random effects were grouped by chip samples to account for the measurements taken from individual chips across multiple stimulation phases. For gene expression array data, average fold‐change (FC) for each gene that has been calculated by averaging (geometric mean) the fold difference (ratio) of relative mRNA expression across all the samples in YA or OS differentiated myobundles compared with YA or OS myoblasts, respectively. Statistical differences between groups were assessed by Student's *t*‐test of replicate raw Ct data between the relative mRNA expression in the differentiated YA and OS cohort compared with undifferentiated YA and OS, respectively.

## AUTHOR CONTRIBUTIONS

S.M, S.G., J.M‐S, and M.P. contributed to conceptualization and data interpretation. S.M, S.G., J.M‐S, M.P., P.M.C., and C.E.S. contributed to experimental research and data analysis. L.K.M, D.P., and P.M.C were involved in providing reagents and materials. L.K.M provided computer‐assisted designs. S.M, S.G., J.M‐S, and M.P. contributed toward writing original draft. All authors contributed to intellectual input, writing review, and editing. S.M provided overall project supervision.

## CONFLICT OF INTEREST

S. Malany is a member of the board at Micro‐gRx, INC. All other authors declare they have no conflicts of interest with the contents of this article.

## Supporting information


Supporting Information S1

Video S1

Video S2

## Data Availability

Data are in the manuscript and the supporting information file. Gene expression raw values are available upon request. Raw and normalized contraction responses and gene expression profiling data are available in the Microphysiological Systems Database available through the University of Pittsburg Drug Discovery Institute https://upddi.pitt.edu/microphysiology‐systems‐database/
