# Supplementary material for: Microphysiological system for studying contractile differences in young, active, and old, sedentary adult derived skeletal muscle cells
Source: Aging Cell. 2022 Jun 2;21(7):e13650. doi: 10.1111/acel.13650 (PMC9282836; doi:10.1111/acel.13650)
Supplement: Supplementary file 1 — Supporting Information S1 [file ACEL-21-e13650-s001.pdf]

Supplemental Material

Microphysiological system for studying contractile differences in young, active and old, sedentary adult derived skeletal muscle cells

Shelby Giza,*[a] Jorge A. Mojica-Santiago*[b], Maddalena Parafati[a], LeGrand K. Malany[c], Don Platt[d], Christine E. Schmidt[b], Paul Coen[e] and Siobhan Malany [#a]

[a]Department of Pharmacodynamics, College of Pharmacy, University of Florida, Florida 32610, USA.

[b]J. Crayton Pruitt Family Department of Biomedical Engineering College of Engineering, Universoty of Florida, Gainesville, Florida 32610, USA

[c]Micro-gRx, INC, Orlando, Florida 32935, USA

[d]Micro Areospace Solutions, Melbourne, Florida 32935, USA

[e]Translational Research Institute, AdventHealth, Orlando, Florida 32804, USA

*Authors contributed equally

[#]Correspondance: Siobhan Malany, Ph.D., Department of Pharmacodynamics, College of Pharmacy, university of Florida, Florida USA. Tel: 407-273-6400, email: smalany@cop.ufl.edu

Running Title: Skeletal Muscle Tissue Chip to model Aging

A

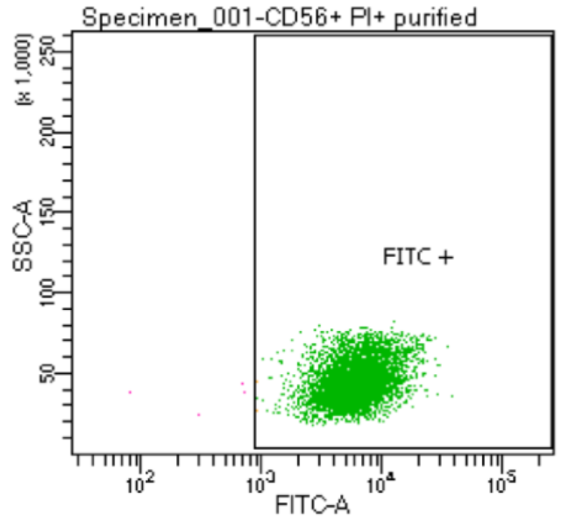

B

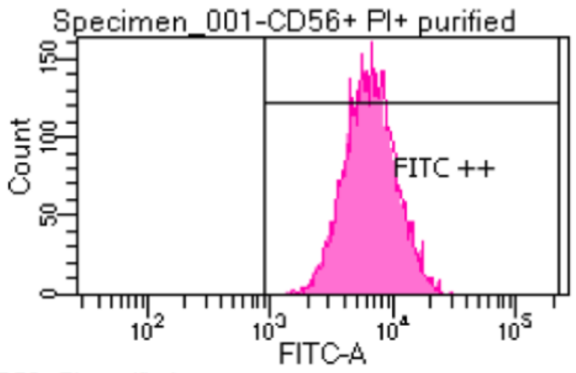

C

Tube: CD56+ PI+ purified

Population	#Events	%Parent	%Total
All Events	10,000	####	100.0
debris	9,436	94.4	94.4
Cells	7,336	77.7	73.4
FSC singles	6,834	93.2	68.3
SSC SINGLES	6,385	93.4	63.8
live cells	4,817	75.4	48.2
FITC +	4,813	99.9	48.1
FITC ++	4,811	99.9	48.1

Figure S1. Isolation and purification of CD56+ Myocytes.

Stained cells were analyzed on a BD FACSCanto II (BD Biosciences, San Jose, CA) equipped with BD FACSDiva software. A.) Dot-plot shows single subpopulation of cells. B.) histogram shows intensity from single florescent channel. C.) Population analysis indicates 99.9% purity for CD56+ population. In brief, purified cells were treated with NA/LE Human Fc Blocker (BD Biosciences, San Jose, CA) to reduce non-specific binding of detection antibodies and incubated on ice with the CD56 antibody clone 5.1H11 prepared in 2% fetal bovine serum in 1X PBS (FACS Buffer) for 30 minutes in the dark. Cells were washed and incubated on ice with a goat anti-mouse IgG (H+L)-Alexa 488 detection antibody prepared in FACS Buffer for 30 minutes in the dark. Cells were washed and stored in 1.5% paraformaldehyde. Specificity of staining was confirmed using a mouse IgG1, kappa-Alexa 488 isotype control antibody.

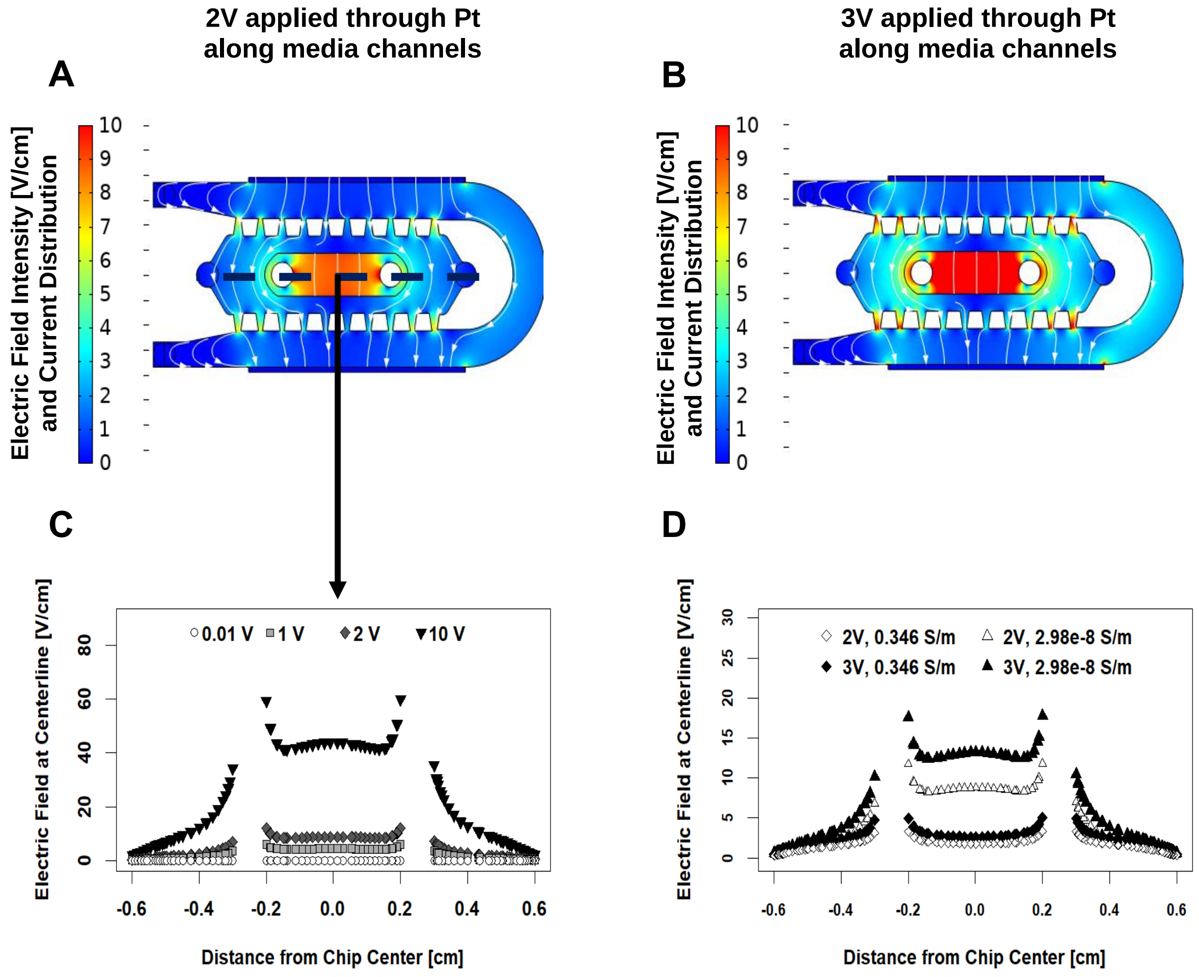

Figure S2. Finite element simulations to determine optimal electric field intensity in the tissue chips.

Color maps indicate differences in the electric field intensity between A) 2V and B) 3V. White arrows show a similar distribution of currents via the electrolytic fluid (cell culture medium) throughout the tissue chip. C) Electric field magnitudes that resulted from a sweep of applied voltage between 10mV and 10V were obtained from the centerlines of the tissue chip bundles and plotted as a function of the distance from the chip center (black dotted line and downward arrow in A. D) A closer inspection of simulations when 2V (white symbols) and 3V (black symbols) were applied with (triangles) and without (diamonds) the tissue chip bundles.

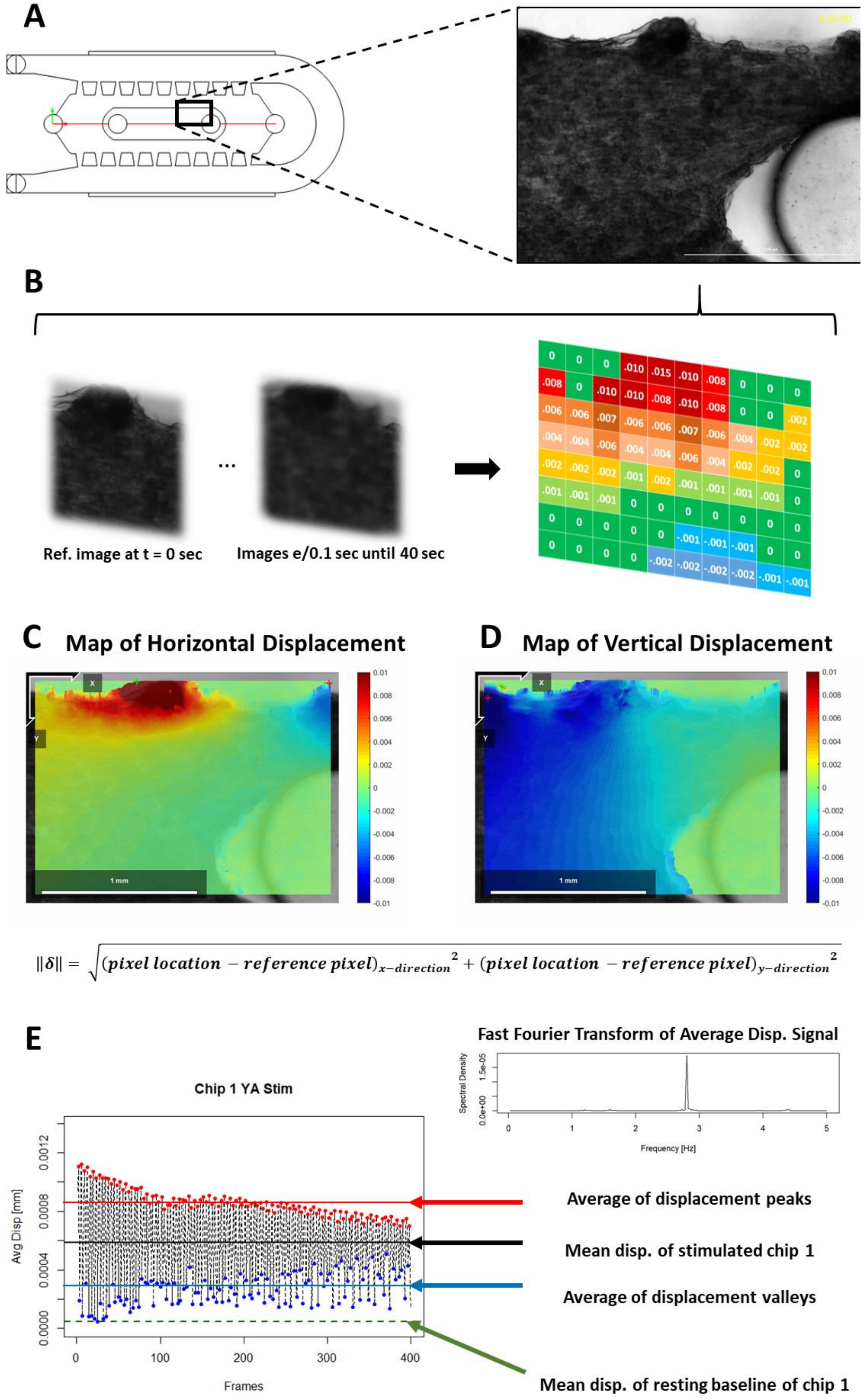

A
B
0 0 0 .010 .015 .010 .008 0 0 0
.008 0 .010 .010 .008 .010 .008 0 0 .002
.006 .006 .007 .006 .006 .007 .006 .004 .002 .002
.004 .004 .006 .004 .004 .006 .004 .002 .002 0
.002 .002 .002 .001 .002 .001 .001 .001 .001 0
.001 .001 .001 0 0 0 0 0 0 0
0 0 0 0 0 -.001 -.001 -.001 0 0
0 0 0 0 -.002 -.002 -.002 -.002 -.001 -.001
Ref. image at t = 0 sec
...
Images e/0.1 sec until 40 sec
C
Map of Horizontal Displacement
D
Map of Vertical Displacement
X
Y
1 mm
0.01
0.008
0.006
0.004
0.002
0
-0.002
-0.004
-0.006
-0.008
-0.01
$$\|\delta\| = \sqrt{(pixel\ location\ - reference\ pixel)_{x-direction}{}^{2} + (pixel\ location\ - reference\ pixel)_{y-direction}{}^{2}}$$
E
Fast Fourier Transform of Average Disp. Signal
Spectral Density
0.0e+00
1.5e-05
0 1 2 3 4 5
Frequency [Hz]
Chip 1 YA Stim
Avg Disp [mm]
0.0000 0.0004 0.0008 0.0012
0 100 200 300 400
Frames
Average of displacement peaks
Mean disp. of stimulated chip 1
Average of displacement valleys
Mean disp. of resting baseline of chip 1

Figure S3. Displacement magnitude acquisition

Workflow of the image analysis used to obtain parameters of contraction from the recorded videos. A) Representative frame indicating the location of a region of interest selected for video recordings of an electrically stimulated myobundle derived from stimulated YA cells. B) Graphic depicting the pixelated image in a sequence of frames from the video were mapped to determine their change in position with respect to a reference image at the beginning of each video, thereby resulting in numerical intensity values (mm) associated to the displacement of each pixel point across the entire region of interest. C) These intensity values were translated to color map plots showing displacements in the axial (horizontal) direction of the myobundle where red pixels correspond to positive contractions from left to right, and D) displacements in the vertical direction of the myobundle where positive contractions are shown from the top to the bottom of the figure (scale bars, 1 mm). The displacements in the axial and vertical components were averaged at each time point to obtain the magnitude of the displacement vector represented as a 40 seconds signal, from which the following parameters were obtained: E) a dominant frequency through a Fast Fourier Transform of the displacement signal, the mean displacement signal of the recorded chip (black), the difference between the average peak displacement (red) and the mean displacement of the signal corresponding to the resting phase of the same chip (green), and the difference between the average valleys in the displacement signal (blue) and the resting phase baseline (green)

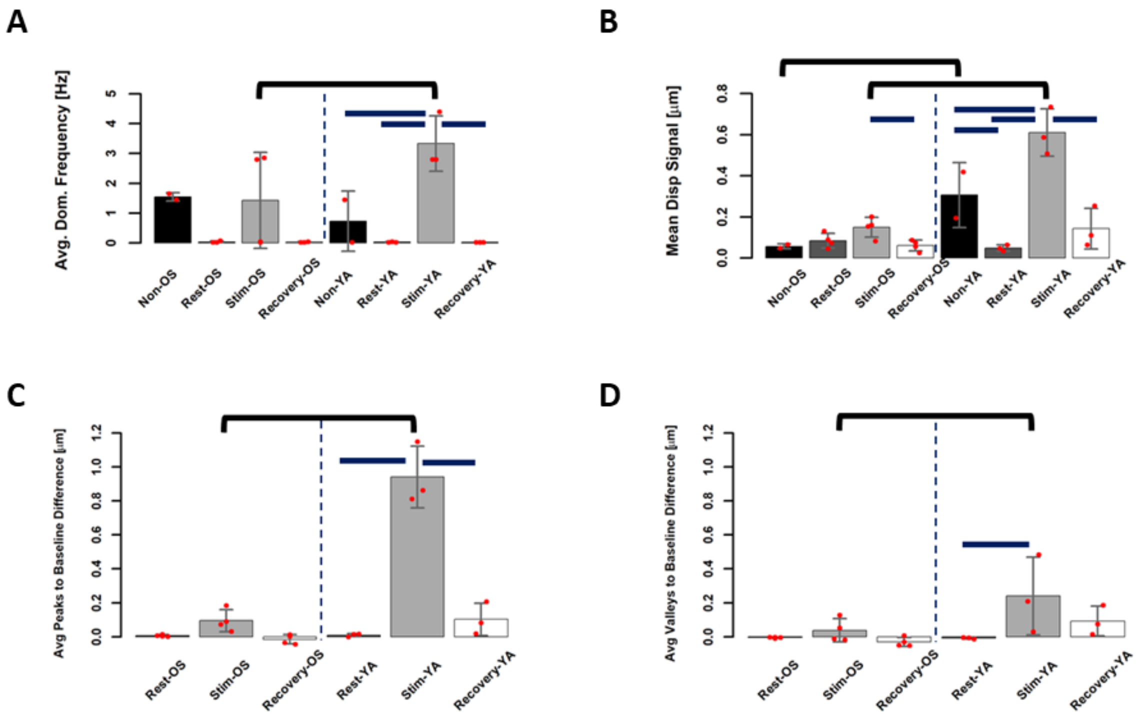

Figure S4. Displacement contraction analysis for 40 sec recording

A-D) The dominant frequency, mean displacement, average peaks above the mean displacement of resting baseline, and average valleys relative difference to the mean displacement of said baseline were extracted from this displacement signal (brackets indicate significant differences between OS and YA-derived myobundles, and solid bars show differences between stimulation phases for individual cell groups at $p < 0.05$.

Range: t = 10-20 sec						
**Sample**	**Cells**	**Treatment**	**Freq. (Hz)**	**Mean Disp. (µm)**	**Peaks to Base (µm)**	**Valleys to Base (µm)**
N = 2	YA	Non-S	1.16	0.13 ± 0.056	N/A	N/A
N = 3	YA	Rest	0.49	0.05 ± 0.01	0.01 ± 0.01	-0.008 ± 0.006
N = 3	YA	Stim	2.78	0.64 ± 0.17	1.00 ± 0.14	0.26 ± 0.27
N = 3	YA	Recovery	0.34	0.12 ± 0.08	0.08 ± 0.09	0.07 ± 0.07
N = 2	OS	Non-S	1.07	0.051 ± 0.004	N/A	N/A
N = 4	OS	Rest	0.56	0.08 ± 0.04	0.01 ± 0.02	-0.009 ± 0.008
N = 4	OS	Stim	1.67	0.15 ± 0.05	0.10 ± 0.07	0.04 ± 0.07
N = 4	OS	Recovery	0.97	0.06 ± 0.02	-0.02 ± 0.03	-0.04 ± 0.03

Range: t = 0-40 sec						
**Sample**	**Cells**	**Treatment**	**Freq. (Hz)**	**Mean Disp. (µm)**	**Peaks to Base (µm)**	**Valleys to Base (µm)**
N = 2	YA	Non-S	0.74	0.31 ± 0.16	N/A	N/A
N = 3	YA	Rest	0.03	0.05 ± 0.02	0.01 ± 0.01	-0.008 ± 0.005
N = 3	YA	Stim	3.33*	0.61 ± 0.12	0.94 ± 0.18	0.24 ± 0.23
N = 3	YA	Recovery	0.03	0.14 ± 0.10	0.10 ± 0.10	0.09 ± 0.09
N = 2	OS	Non-S	1.55	0.06 ± 0.01	N/A	N/A
N = 4	OS	Rest	0.04	0.08 ± 0.04	0.007 ± 0.006	-0.005 ± 0.003
N = 4	OS	Stim	1.43	0.15 ± 0.05	0.10 ± 0.06	0.04 ± 0.07
N = 4	OS	Recovery	0.03	0.06 ± 0.03	-0.02 ± 0.03	-0.03 ± 0.03

Figure S5. Summary of the parameters extracted from the displacement signal at a subset of the time range (top table) and the full-time range (bottom table).

N (Number of chips with myobundles); YA (young athletic); OS (old sedentary); Non-S (non-stimulated chips); Rest (chip baseline at resting phase before electrical stimulation); Stim (phase during electrical stimulation); Recovery (recovery phase after electrical stimulation); Freq. (dominant frequency of the peaks in the displacement signal); Mean Disp. (average of the displacement signal); Peaks to Base (average difference between local maxima of displacement signal and resting phase baseline); Valleys to Base (average difference between the local minima of displacement signal and resting phase baseline). *A dominate peak occurs at 4 Hz due to spontaneous contraction in addition to the peak at 2.8 Hz providing an average of 3.33 Hz.

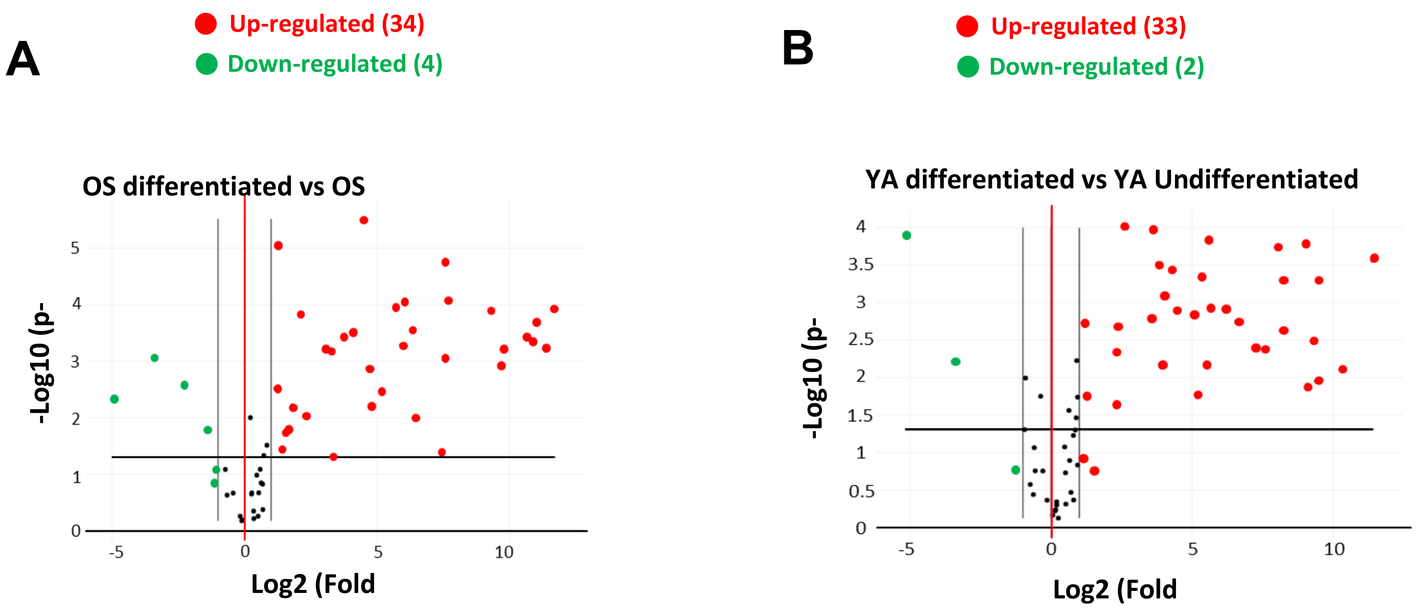

Figure S6. Differentially expressed genes between the YA and OS cohorts. Volcano plot visualizing skeletal myogenesis and muscle contractility genes that differed between differentiated OS vs undifferentiated OS (A) and differentiated YA vs undifferentiated YA (B). The x-axis represents the mean of log2 fold-change (FC) value, and the yaxis corresponds to the negative logarithm of the P-values. The horizontal line represents the level of significance for the t tests performed p-value ≤0.05, and the vertical lines display the threshold set log2 fold change (FC)≥2. The red and green circles show genes that were significantly up- or down-regulated and the black circles show genes that were not significantly changed in our 3D contractile skeletal muscle tissues. Genes outside the two black vertical lines are up- or downregulated more than 2-fold. On the y-axis, the –log10 of the P value is plotted; thus, the higher values indicate stronger statistical evidence of a significant difference in gene expression between YA/OS differentiated vs YA/OS undifferentiated.

	YA No EStim		OS No EStim		YA EStim		OS EStim	
Gene	FC	p Value	FC	p Value	FC	p Value	FC	p Value
ACTA1	546.45	0.01263	1667.97	0.000385	1009.52	0.000031	1380.48	0.000979
ACTN3	147.78	0.004	60.79	0.000519	314.43	0.001088	48.22	0.000345
ADRB2	5.16	0.00208	1.45	0.214751	1.28	0.567232	1.64	0.28641
AGRN	1.42	0.18557	1.28	0.615233	2.19	0.022409	1.35	0.504966
ATP2A1	192.09	0.004371	87.6	0.010354	547.72	0.012655	84.71	0.000002
BCL2	15.13	0.007213	6.79	0.067422	14.1	0.00472	2.64	0.584068
BMP4	1.74	0.427652	2.3	0.161662	2.54	0.078551	1.51	0.097217
CAMK2G	5.16	0.004524	3.05	0.016751	6	0.000878	3.87	0.020168
CAPN2	0.52	0.049335	0.49	0.085215	0.58	0.087115	0.54	0.121039
CAPN3	36.6	0.01802	27.61	0.006046	95.02	0.009761	29.5	0.00237
CAST	1.11	0.594376	0.89	0.560154	1.86	0.067026	0.99	0.908216
CAV1	0.09	0.005888	0.1	0.000861	0.03	0.004639	0.12	0.000891
CAV3	41.17	0.000474	53.16	0.000108	69.85	0.000131	52.6	0.000043
CRYAB	0.6	0.265166	0.47	0.146318	0.69	0.436896	0.5	0.180922
CS	1.88	0.005939	1.8	0.030889	2.5	0.006875	1.73	0.08515
CTNNB1	0.91	0.429244	1.66	0.047123	2.2	0.005644	1.23	0.495286
DAG1	1.86	0.034309	2.33	0.000009	2.56	0.000908	2.33	0.035113
DES	13.86	0.000305	17.27	0.000299	16.44	0.000224	20.86	0.002922
DMD	50.38	0.001177	67.6	0.00009	62.41	0.003875	75.19	0.000965
DMPK	19.13	0.000383	22.77	0.000003	18.76	0.000218	24.71	0.000282
DYSF	34.15	0.001513	25.25	0.001332	41.06	0.003734	23.41	0.010006
HDAC5	1.15	0.498212	1.54	0.143538	1.54	0.012179	1.7	0.039599

**HK2**	0.66	0.085875	0.74	0.217708	0.62	0.287228	0.62	0.307599
**IGF1**	2.08	0.116939	2.71	0.036122	3.72	0.047994	2.98	0.104289
**IGFBP3**	0.42	0.168032	0.21	0.002542	0.38	0.031588	0.27	0.003601
**IGFBP5**	46.54	0.007061	36.45	0.003414	80.65	0.001717	35.07	0.024072
**IKBKB**	1.81	0.049902	1.51	0.082492	2.6	0.012081	1.73	0.042213
**LMNA**	0.68	0.174435	1.27	0.453218	1.96	0.092498	1.01	0.982001
**MAPK1**	1.58	0.127062	1.61	0.151176	0.89	0.579851	1.61	0.147584
**MB**	682.55	0.011198	173.37	0.044008	1775.22	0.000631	181.94	0.000019
**MEF2C**	47.34	0.000151	81.09	0.000285	59.11	0.012462	73.52	0.00112
**MSTN**	16.08	0.000788	8.98	0.000629	39.5	0.000047	7.64	0.062875
**MUSK**	1.63	0.337981	2.98	0.017287	2.01	0.056947	1.86	0.147048
**MYF5**	0.03	0.000125	0.03	0.004836	0.07	0.000163	0.04	0.005708
**MYF6**	6.36	0.000101	4.35	0.000149	7.93	0.000028	3.11	0.009766
**MYH1**	1269.86	0.007972	3363.41	0.000118	1066.46	0.002764	2517.22	0.000281
**MYH2**	74.15	0.001239	192.03	0.000859	145.54	0.039158	141.37	0.000038
**MYOD1**	12.15	0.000114	14.15	0.00037	13.73	0.006482	15.47	0.010582
**MYOG**	99.61	0.001853	614.26	0.000124	137.87	0.010748	550.51	0.000667
**MYOT**	268.07	0.000188	189.5	0.000018	210.69	0.001416	170.91	0.000388
**NEB**	642.68	0.003262	2041.91	0.000195	803.85	0.004263	1587.03	0.00425
**PAX3**	1.44	0.48429	1.43	0.55943	1.52	0.428281	2.01	0.12934
**PAX7**	0.89	0.607706	1.25	0.827224	1.51	0.7125	1.24	0.754623
**PDK4**	2.87	0.167902	0.37	0.016949	4.4	0.114709	0.21	0.010162
**PPP3CA**	1.15	0.451153	1.21	0.216195	1.62	0.010968	1.24	0.208169
**RHOA**	0.77	0.017737	1.2	0.22603	0.93	0.634339	1.09	0.473666
**RPS6KB1**	2.26	0.001956	2.26	0.003044	3.55	0.01519	2.25	0.015407

**SGCA**	2.37	0.017339	3.49	0.006224	3.3	0.004231	3.12	0.003581
**SLC2A4**	23.19	0.001256	10.13	0.048677	32.7	0.070305	15.86	0.020883
**TNNC1**	536.69	0.000175	809.12	0.001183	680.11	0.001501	789.4	0.001708
**TNNI2**	2724.2	0.000273	1848.8	0.000419	5702.12	0.003553	1516.59	0.000603
**TNNT1**	306.3	0.000522	213.34	0.000083	469.65	0.008944	201.75	0.003249
**TNNT3**	714.25	0.000504	2627.01	0.000579	1306.01	0.001316	2928.51	0.001745
**TTN**	307.71	0.002489	870.81	0.000601	309.85	0.002942	626.98	0.001489
**UTRN**	4.93	0.024306	4.82	0.009205	7.51	0.001765	5.22	0.029636

Figure S7: Gene expression differences between differentiated (No E-Stim and E-Stim) vs undifferentiated cohorts. Average fold-change (FC) for each gene that has been calculated by averaging (geometric mean) the fold difference (ratio) of relative mRNA expression across all the samples in YA or OS differentiated myobundles compared to YA or OS myoblasts, respectively. Therefore, values higher than 1 indicate up-regulation while values lower than 1 indicate down-regulation. P-values correspond to student t-test of replicate raw Ct data between the relative mRNA expression in the differentiated YA and OS cohort compared to undifferentiated YA and OS, respectively.

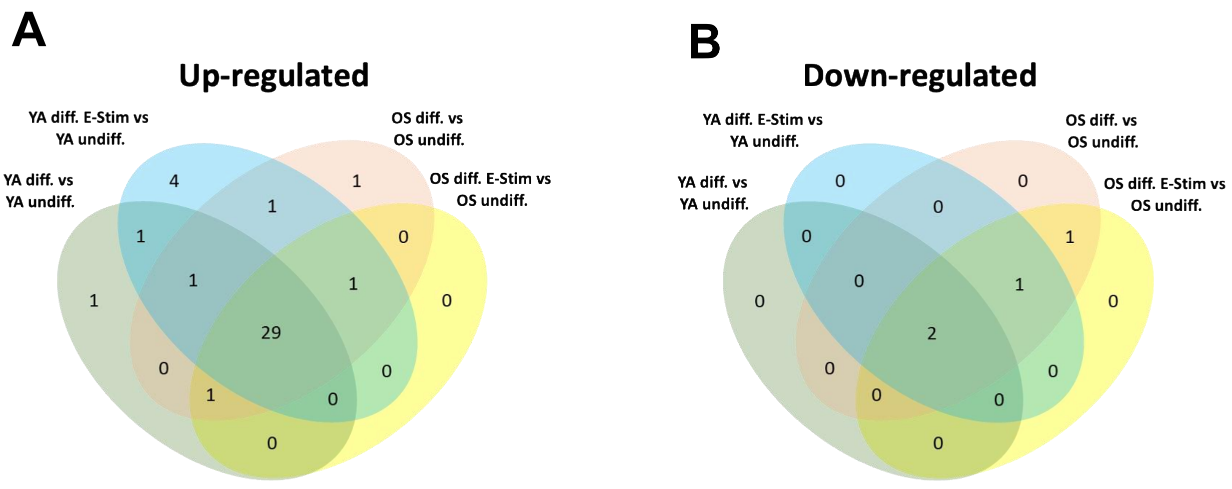

Figure S8. Venn Diagram illustrating the results of A) up- and B) down-regulated differential gene expression analysis (YA vs OS) in various groups of samples: YA E-Stim, YA no E-Stim, OS E-Stim, OS no E-Stim. In Venn diagrams, we included the numbers of shared and specific DEGs between groups that passed thresholds: fold change > 2 and with $p < 0.05$.

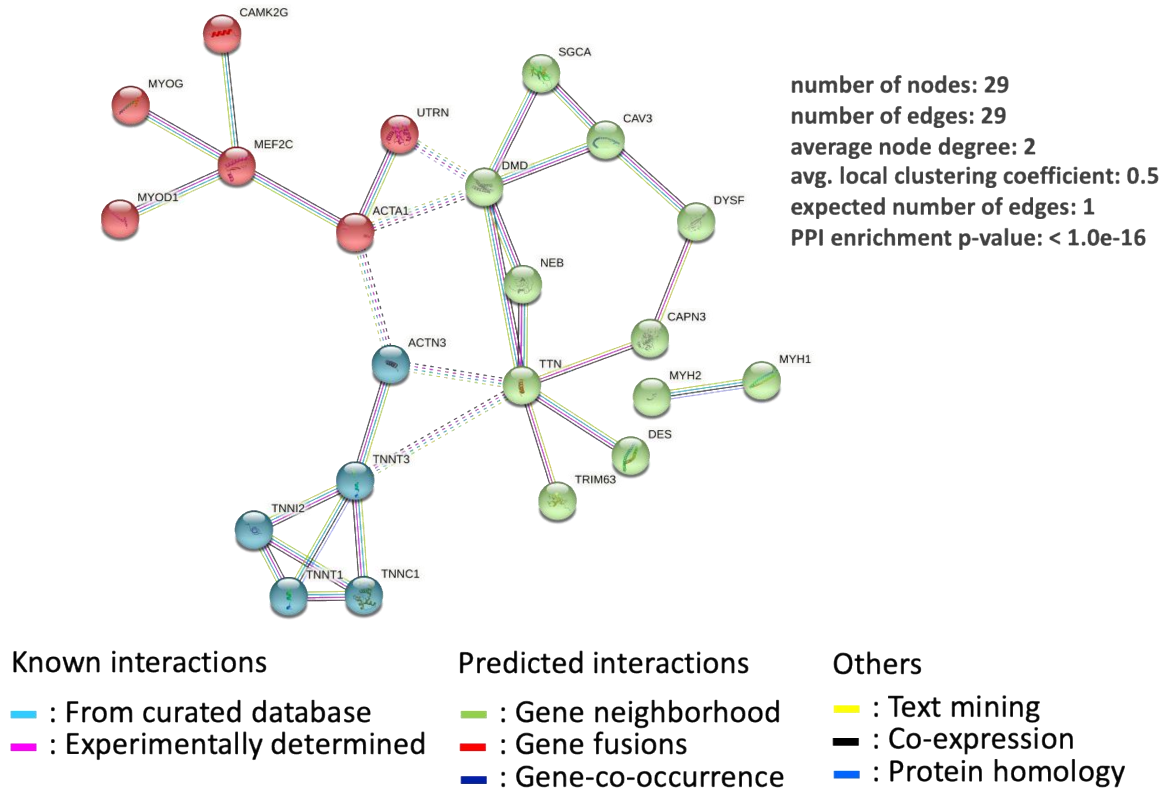

Figure S9. Protein interaction networks generated based on DEG in YA and OS derived myobundles. Protein interaction networks, generated using STRING database analysis tool (https://string-db.org/) for the top 29 genes that were substantially overexpressed YA and OS derived myobundles with or without E-Stim, identified 3 clusters shown in red, green and blue. Only proteins with at least one interaction are shown. Subcellular localization and biological process are annotated. We applied the KMEANS clustering algorithm to cluster the proteins displayed in the network.

Gene ontology

GO-term	description	false discovery rate
**Biological process**		
GO:0003012	muscle system process	1.13E-34
GO:0006936	muscle contraction	2.01 e-27
GO:0030049	muscle filament sliding	1.39 e-18
GO:0090257	regulation of muscle system process	4.58 e-18
GO:0043502	actin mediated cell contraction	2.85 e-17
**Molecular function**		
GO:0008092	cytoskeletal protein binding	4.30 e-13
GO:0008307	structural constituent of muscle	2.12 e-7
GO:0003779	actin binding	8.35 e-7
GO:0005515	protein binding	4.45 e-5
GO:0031014	troponin T binding	0.00011
**Cellular component**		
GO:0030016	myofibril	2.65 e-22
GO:0030017	sarcomere	2.24 e-21
GO:0099512	supramolecular fiber	8.10 e-16
GO:0031674	I band	2.47 e-10
GO:00015629	actin ctoskeleton	5.53 e-10

Figure S10. Top GO terms identified based on DEG in YA and OS derived myobundles with or without E-stim. GO terms for biological process, molecular function and cellular component are listed.
